# Single-cell diploid Hi-C reveals the role of spatial aggregations in complex rearrangements and KMT2A fusions in leukemia

**DOI:** 10.1186/s13059-022-02740-9

**Published:** 2022-08-09

**Authors:** Zhihao Xing, Huirong Mai, Xiaorong Liu, Xiaoying Fu, Xingliang Zhang, Lichun Xie, Yunsheng Chen, Adam Shlien, Feiqiu Wen

**Affiliations:** 1 Clinical Laboratory & Division of Hematology and Oncology, Shenzhen Children’s Hospital, Shenzhen, China; 2 Program in Genetics and Genome Biology, The Hospital for Sick Children, Toronto, ON Canada; 3 Shenzhen Institute of Pediatrics, Shenzhen Children’s Hospital, Shenzhen, China; 4 Division of Hematology and Oncology, Shenzhen Children’s Hospital, Shenzhen, China

**Keywords:** Single-cell Hi-C, Fusions, Leukemia, Genomic rearrangements, Complex rearrangements

## Abstract

**Background:**

Simple translocations and complex rearrangements are formed through illegitimate ligations of double-strand breaks of fusion partners and lead to generation of oncogenic fusion genes that affect cellular function. The contact first hypothesis states that fusion partners tend to colocalize prior to fusion in normal cells. Here we test this hypothesis at the single-cell level and explore the underlying mechanism.

**Results:**

By analyzing published single-cell diploid Hi-C datasets, we find partner genes fused in leukemia exhibit smaller spatial distances than those fused in solid tumor and control gene pairs. Intriguingly, multiple partners tend to colocalize with KMT2A in the same cell. 3D genome architecture has little association with lineage decision of KMT2A fusion types in leukemia. Besides simple translocations, complex rearrangement-related KMT2A fusion genes (CRGs) also show closer proximity and belong to a genome-wide mutual proximity network. We find CRGs are co-expressed, co-localized, and enriched in the targets of the transcriptional factor RUNX1, suggesting they may be involved in RUNX1-mediated transcription factories. Knockdown of RUNX1 leads to significantly fewer contacts among CRGs. We also find CRGs are enriched in active transcriptional regions and loop anchors, and exhibit high levels of TOP2-mediated DNA breakages. Inhibition of transcription leads to reduced DNA breakages of CRGs.

**Conclusions:**

Our results demonstrate KMT2A partners and CRGs may form dynamic and multipartite spatial clusters in individual cells that may be involved in RUNX1-mediated transcription factories, wherein massive DNA damages and illegitimate ligations of genes may occur, leading to complex rearrangements and KMT2A fusions in leukemia.

**Supplementary Information:**

The online version contains supplementary material available at 10.1186/s13059-022-02740-9.

## Introduction

Genomic rearrangements (structural variations) have been discovered to play a role in tumors [1, 2], which can affect cellular functions by generating abnormal fusion genes. Genomic rearrangements can be classified into simple rearrangements (including translocations, inversions, deletions, and duplications) and complex rearrangements (CRs) (including chromoplexy and chromothripsis). CRs have been recently found in 5–9% of tumor genomes and implicated in tumorigenesis [1, 3]. In leukemia, more than 10.5% of KMT2A (MLL) fusions result from CRs [4]. Fusion occurs when two previously independent genes are placed side by side, which usually originate from translocations [5]. Gene fusions are frequently associated with carcinogenic properties and are driver mutations in various cancers [6, 7]. More than 300 frequent fusions have been identified in hematological disorders and malignant solid tumors. However, the molecular process of generating oncogenic fusions, especially CRs, remains poorly understood [8].

The formation of fusions is a multistep process, including DNA double-strand breaks (DSBs), spatial proximity, and illegitimate DNA ligation. NHEJ is reported to be responsible for generations of fusions in multiple cancers [9]. Since illegitimate DNA ligation of two DSBs requires spatial proximity, the “contact first” hypothesis was proposed, which states that in normal cells, genomic fusion partners tend to colocalize prior to fusion [10]. However, it remains unclear to what extent three-dimensional (3D) spatial organization contributes to fusions in human hematologic malignancies [10].

Recent developments of 3C techniques such as bulk Hi-C have substantially advanced the studies of fusions. Hi-C sequences experimentally ligated proximate DNA fragments in 3D space and can detect 3D chromatin structures at the genome-wide level [11, 12]. This technique is superior to FISH (Fluorescence in situ hybridization) in terms of resolution and throughput [13]. With Hi-C, it was revealed that the frequencies of fusions in mouse pro-B cell line were proportional to the spatial distances of the fusion partner genes (measured with the number of Hi-C contacts) in normal cells [14], which provided the first genome-wide evidence that 3D chromatin structures may influence the genomic fragments subject to translocation in tumors. Further, applying the Hi-C approach on human cell lines demonstrated that 3D chromatin structures could shape the landscapes of translocations which often result in oncogenic fusions [15]. Nevertheless, bulk Hi-C presents an average ensemble of all 3D chromatin structures in a cell pool [12], so it is hard to provide the chromatin organizations in a single cell. Possibly, a tumor may originate from a single mutated cell, so to capture the chromatin organization of a tumor precursor cell can provide clues on the process of oncogenic fusions. Excitingly, single-cell Hi-C was developed and solves this problem [16]. With single-cell Hi-C, one can measure the dynamic spatial locations of many genes in a single cell. Single-cell Hi-C has been applied in both haploid mouse cells [17] and diploid human cells [18], providing great insights into the chromatin organizations.

In this study, we take advantage of the recently published single-cell Hi-C data that consist of 29 blood cells to explore the formation mechanism of oncogenic fusions in leukemia. The 29 cells comprise several types of blood cells, such as lymphocytes and myelocytes, providing an opportunity to investigate tumor precursor cells of different leukemia subtypes.

## Results

### Single-cell diploid Hi-C data reveals that leukemia fusion partner genes are spatially closer than expected in normal human blood cells

To start with, we collected 297 oncogenic fusions from the COSMIC database [19], of which 92 are intra-chromosomal fusions and 205 are inter-chromosomal ones (Additional file 2: Table S1). Here we restrict our following analyses into inter-chromosomal fusions only for the following reasons: (1) many intra-chromosomal fusions involve genes in close linear genomic distance, and the Hi-C accuracy of such close gene pairs is low [12], and (2) most of the leukemia fusions (the focus of this study) are inter-chromosomal, so our results should be representative. In this way, a total of 58 leukemia inter-chromosomal fusions were obtained (Additional file 2: Table S1), of which 45 (78%) involve partner gene KMT2A. In addition, four fusions are associated with ETV6, namely ETV6-ABL1, ETV6-JAK2, ETV6-NTRK3, and ETV6-RUNX1. Most fusion partners seem to be evenly distributed on each chromosome (Additional file 1: Fig. S1A). And the number of fusion genes on a chromosome positively correlates with the total number of genes on the chromosome (Additional file 1: Fig. S1B), suggesting that inter-chromosomal fusions are largely random.

**Fig. 1:**
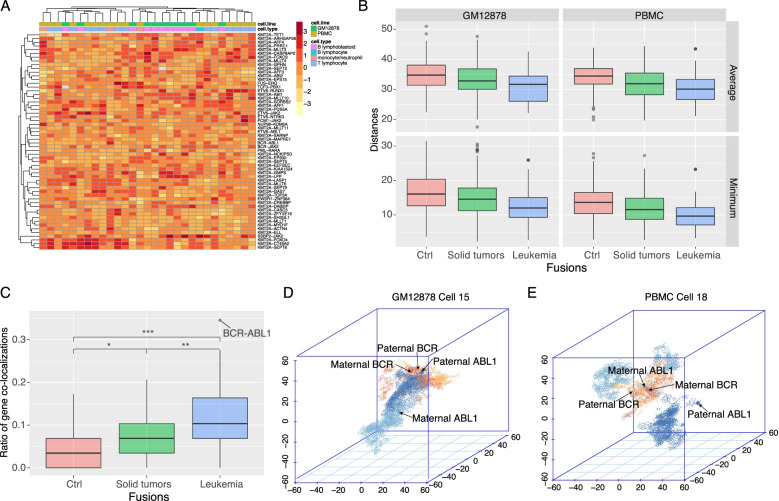
Single-cell Hi-C revealed that leukemia fusion partner genes are closer to each other in the nucleus. **A** Heatmap of spatial distances of different fusion gene pairs across single cells. Rows represent fusions, and columns represent different cells. **B** Comparison of average and minimum Euclidean distance (EuD) values of fusion gene pairs between leukemia, solid tumors, and control. **C** Comparison of the colocalization ratios of fusion partners between leukemia, solid tumors, and controls. **D**, **E** The spatial locations of BCR and ABL1 alleles in the 3D nucleus of two single cells (GM12878 cell 15 and PBMC cell 18), both paternal and maternal alleles, are marked. The statistical tests in **B** and **C** are Wilcoxon rank-sum test

With the single-cell diploid Hi-C data, we first observed that the spatial distances of the leukemia fusion partner genes vary among cells and that the cells of the same type tend to cluster together (Fig. 1A). Here, the distance of a gene pair is defined as the minimum of two alleles (one from each gene), regardless of parental origins. Based on the contact first hypothesis, we hypothesize that the genes involved in oncogenic fusions may be close in space before the actual fusions occur. To test this hypothesis, we classified genes into three groups according to their tumor status in the COSMIC database [19]: genes involved in leukemia fusions, genes involved in solid tumor fusions, and other genes as control (see the “Methods” section). Then for each fusion gene pair, using the single-cell blood Hi-C data, we calculated the average and minimum EuD values over all single cells. As expected, we found that leukemia fusion genes exhibit significantly higher proximities than the solid tumor fusion genes, and the latter show higher proximities than the controls, in both GM12878 and PBMC cell lines (Fig. 1B). These results are consistent with the prediction of the contact first hypothesis. Since the Hi-C data are derived from blood cells, they may represent the precursor cells of leukemia better than the precursors of solid tumors, so leukemia fusion genes show higher proximities than solid tumor fusion genes in the data, and we expect the relationship reverses if Hi-C data from tissues related to solid tumor are used. Figure 1B illustrates that the average EuD values of more than 75% of fusion partner genes are less than 35 (around 3.5μm), consistent with previous reports based on FISH [20].

Alternatively, we defined co-localization ratio as the ratio of the count of single cells in which two genes are co-localized to the count of all single cells. Comparing the ratios among the gene groups, leukemia fusion partners have significantly higher colocalization ratios than solid tumor fusion partners and controls (Fig. 1C). Philadelphia chromosome (Ph) fusion BCR-ABL1 (colocalization ratio=34.9%) and KMT2A-ELL (colocalization ratio=27.6%) are the top 2 fusions with the highest colocalization ratios (Fig. 1C). As an example, the BCR and ABL1 locations in the nuclei of GM12878 cell 15 and PBMC cell 18 are shown in Fig. 1D and E.

As a supplementary analysis, we also used the gene fusions from the database TumorFusions [21], from which we obtained 62 leukemia gene fusions. Thirty-four of the fusions are inter-chromosomal and used here (Additional file 2: Table S1). Consistent with the above observation, we found that leukemia fusion gene pairs exhibit higher proximities than the control gene pairs (Additional file 1: Fig. S2).

### KMT2A fusion partner genes are spatially proximate to the gene KMT2A

**Fig. 2:**
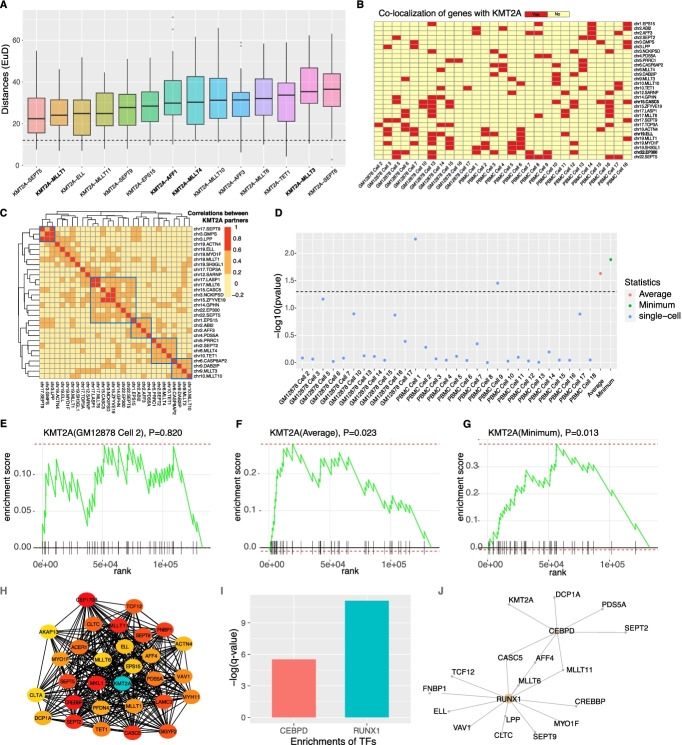
The 3D spatial structures between KMT2A and fusion partners. **A** The boxplots of Euclidean distances among single cells between KMT2A and its 14 closest fusion partners. **B** The co-localizations of KMT2A and different fusion partners in each cell. **C** The colocalization correlations across cells between different KMT2A partners. **D** The *P*-values computed from the comparisons of the distances to KMT2A between partner genes and the other genes in a single cell as well as the average and minimum distances among the groups. **E**–**G** GSEA results of GM12878 Cell 2 and average, minimum of all single-cells. **H** The colocalization network of the top 30 KMT2A fusion partners which have the largest numbers of colocalized partners. A darker color for a node (except KMT2A gene, which is highlighted by green) means more connections in the network. **I** Transcriptional enrichments of the top 30 KMT2A fusion partners using Enrichr. **J** Most of the top 30 partners are the targets of transcription factors RUNX1 and CEBPD

KMT2A fusions account for 78% of fusions in leukemia and the frequencies of different forms vary from 0.05 to 35% (Additional file 2: Table S1), suggesting that KMT2A fusions may be an important driver of leukemia. A previous study of the frequent KMT2A fusion partner genes based on the FISH technique revealed that the spatial distance of MLLT1 to KMT2A is much closer than the distances of the other three partner genes to KMT2A: AFF1, MLLT4, and MLLT3 [20]. Using the Hi-C data, we confirmed the finding (Additional file 1: Fig. S3A-B, ANOVA test: *P*=0.0107; Fig. 2A), suggesting that the Hi-C data are of pretty high quality.

Based on the spatial distances between KMT2A and fusion partner genes, we identified top 14 partners with the smallest median distances (Fig. 2A). Using the FISH technique, we verified that the gene pair KMT2A-ELL indeed has closer spatial distance than KMT2A-MLLT3 (Additional file 1: Fig. S3C-D), suggesting that the Hi-C results are reliable. Next, we present the fusion partner genes colocalized with KMT2A in each cell, as shown in Fig. 2B and C. Despite high heterogeneities across 29 single cells, more than one gene may colocalize with KMT2A simultaneously in a single cell, such as MLLT3 (chr9) and MLLT10 (chr10) in PBMC cell 10 (Fig. 2B, C). Genes on the same chromosomes may show different colocalization patterns (Fig. 2B).

Next, we test whether the spatial distances of the partner genes to KMT2A are smaller than those of the other genes to KMT2A in each single cell. Using the GSEA enrichment strategy (see the “Methods” section), we found no difference between the partner genes and the other genes (Fig. 2D–G). However, we see the partner genes are significantly closer to KMT2A when the average or minimum distance over all single cells is used (Fig. 2D–G). The disparity between the bulk and single-cell levels has been seen before: for example, few Nanog-partner interactions identified using bulk 4C can be confirmed in single cells [16]. The disparity may be explained by the heterogeneity of the colocalizations of partner genes (Fig. 2B): in a single cell, only a few of all the partner genes colocalize with KMT2A, so one may not see a closer distance to KMT2A when all the partner genes are considered.

Next, we ask whether the KMT2A partner genes are enriched in the targets of some transcriptional factors. To do so, we constructed a colocalization network by choosing partners whose distances to KMT2A are EuD<15 in at least three single cells, and then selected the top 30 partner genes with the most connections (Fig. 2H). Using Enrichr tool and the datasets CHEA and ENCODE [22], we found that these partner genes are significantly enriched in the targets of transcriptional factors CEBPD and RUNX1 (Fig. 2I, J).

**Fig. 3:**
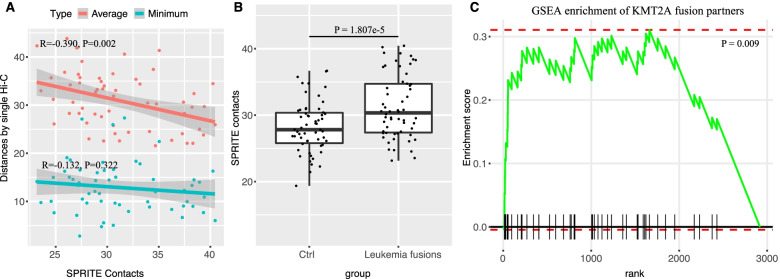
SPRITE verified that leukemia fusion partners are in close proximity. **A** Correlation between the spatial distances of fusion partners measured using single-cell Hi-C and the number contacts measured using SPRITE (more contacts mean closer spatial distance). For the Hi-C data, both average and minimum values over single cells are used. **B** Fusion partners exhibit higher numbers of SPRITE contacts than control (Wilcoxon rank-sum test). **C** GSEA results indicate that KMT2A partners are significantly enriched in regions closer to KMT2A (*P*-value=0.009)

Our results are further confirmed by using the SPRITE dataset [23]. SPRITE was similar to Hi-C, but it bypasses the step of ligation and can detect farther inter-chromosomal contacts [23]. Also, partner genes in closer proximity show more SPRITE contacts. First, we see a negative correlation between the average EuD values from the single-cell Hi-C and the number of SPRITE contacts for all leukemia fusion partner genes, though not significant when minimum EuD values were used (Fig. 3A). With the SPRITE data, we confirmed that fusion partner gene pairs are spatially closer than control gene pairs (Fig. 3B, Mann-Whitney *U* test, *P*=1.807e-5) and that partner genes are significantly closer to KMT2A overall (Fig. 3C, *P*=0.009). These results further support that the spatial proximity may play an important role in forming fusion genes.

### 3D genome structure has no association with the gene fusions specific to the subtypes of leukemia

**Fig. 4:**
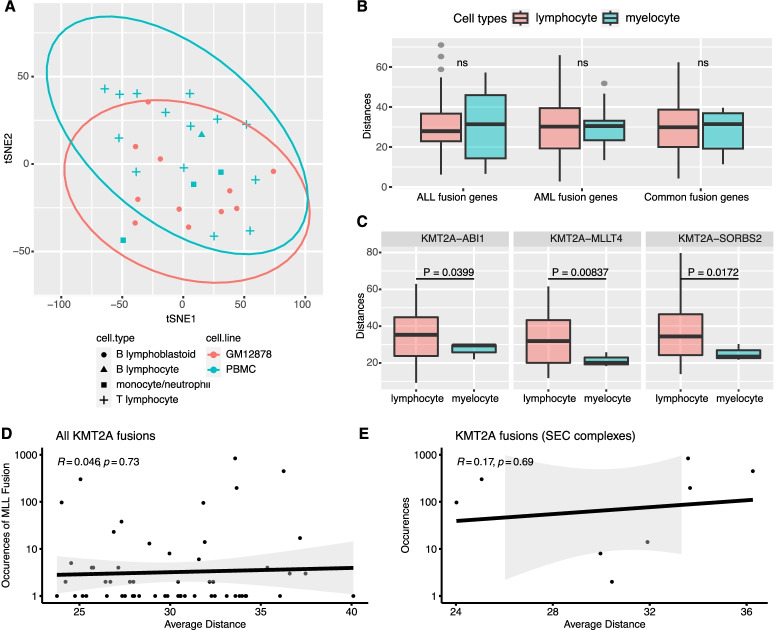
The relationship between 3D genome structures and lineage-specific KMT2A fusions. **A** PCA results of all single cells based on the Euclidean distances between KMT2A and fusion partners. **B** Comparison of KMT2A-partner Euclidean distances between in lymphocytes and in myeloid cells, for AML-specific, ALL-specific fusions, and common fusions. **C** Comparisons for three AML-specific fusions which exhibit closer proximities in myeloid cells than in lymphocytes. **D** The relationship between the KMT2A-partner Euclidean distances and the prevalence of each fusion. **E** The same as **D**, but consider only partner genes belonging to SEC complex

We next examined whether 3D genome structures contribute to the cell-type specific fusions and in turn the tumor types. First, using the KMT2A-partner distances in each cell as variables, the cells under study appear clustered based on cell types: B lymphoblastoid cells, T lymphocytes, and myelocytes (Fig. 4A). Next, we compared the spatial distances of the KMT2A-partner gene pairs between lymphocytes and myelocytes. Assuming that lymphocytes and myelocytes are similar to the precursors of acute lymphoblastic leukemia (ALL) and acute myeloid leukemia (AML), respectively, we might expect that ALL-specific fusion gene pairs have smaller distances in lymphocytes than in myelocytes, and *vice versa* for AML-specific fusion gene pairs. To test this, we grouped the fusion gene pairs based on their tumor sources: ALL-specific, AML-specific, and common to ALL and AML, and then for each group, we compared their spatial distances of partner gene pairs between in lymphocytes and in myelocytes. Overall, we found no significant difference for any group of gene pairs (Fig. 4B). We also found no differences for gene pairs involved in AML or ALL most prevent gene fusions (Additional file 1: Fig. S4A). However, we found three fusion gene pairs exhibited closer distances in myelocytes than in lymphocytes (Fig. 4C): KMT2A-ABI1 (*P*=0.0399), KMT2A-MLLT4 (*P*=0.00837), and KMT2A-SORBS2 (*P*=0.0172). Intriguingly, all these three fusions are exclusively found in AML [4].

Further, we examined this issue in the ALL subtypes: T-cell ALL (T-ALL) and B-cell ALL (B-ALL). The most frequent KMT2A partners for B-ALL are AF4 (57%) and ENL (~18%), while the ones for T-ALL are MLLT4 (39%) and MLLT1 (37%) [4]. Again, we do not see any significant difference for these frequent fusions when comparing the spatial distances between T and B lymphocytes (Additional file 1: Fig. S4B). These results suggest that the cell-type specific 3D genome structures have no association with the leukemia subtype specific fusions.

Here we also measure the prevalence of each KMT2A fusion using a dataset of 2345 leukemia patients [4] and test whether the gene pairs of more prevalent fusions are in closer proximity than the pairs of less prevalent fusions. With the dataset, each fusion’s occurrence was counted over all the patient genomes, with prevalent fusions having larger counts. As shown in Fig. 4D, we do not see significant correlation between fusion prevalence and the spatial distance of partner genes (*R*=0.046, *P*=0.73). One caveat of our analysis here is that certain fusions may be favored in tumors because of their functions. Therefore, as a control, we consider only genes of the SEC complex components (AFF1, AFF2, AFF4, MLLT3, MLLT1, MLLT10, MLLT6, and ELL), assuming the fusions formed between each of these genes and KMT2A have similar functions. Again, no correlation is observed between the fusion prevalence and the spatial distance of partner genes (Fig. 4E, *R*=0.17, *P*=0.69). These results suggest that the prevalence of fusions in leukemia is not associated with the spatial distance of partner genes in precursor cells.

### Complex rearrangement-related genes (CRGs) from KMT2A fusions colocalize with KMT2A and partner genes

**Fig. 5.**
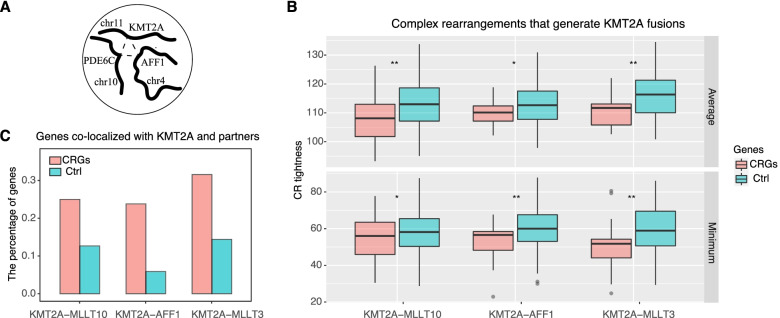
KMT2A fusion complex rearrangement related genes (CRGs) are colocalized with KMT2A and partner genes better than control. **A** An example of complex rearrangements (CRs) involving KMT2A, AFF1, and PDE6C. **B** Comparisons of CR tightness between CRGs and control for three fusions KMT2A-AFF1, KMT2A-MLLT3, and KMT2A-MLLT10. The smaller the CR tightness score, the closer the genes. **C** CRGs are more often colocalized with KMT2A and fusion partners simultaneously than control genes; the *y*-axis shows the percentage of genes that are co-localized with KMT2A and fusion partners simultaneously

In addition to the simple fusions involving two genes, KMT2A fusions can arise from complex rearrangements (CRs), which involve other passenger genes besides KMT2A and fusion partners [3] (such as PDE6C in Fig. 5A). We call these passenger genes complex rearrangement-related genes (CRGs). Single-cell Hi-C is a powerful tool to study CRs involving multiple chromosomal loci. First, we obtain KMT2A fusion-related CRGs from a previous study [4]. Most of them are related to the fusions KMT2A-AFF1, KMT2A-MLLT3, or KMT2A-MLLT10 [4]. We defined a quantity termed “CR tightness” to measure to what extent CRGs are tightly located with KMT2A and partner genes in 3D nuclei (see the “Methods” section). The smaller the “CR tightness,” the closer to KMT2A and partners the CRG is. Despite high cellular heterogeneity, we found that CRGs exhibited smaller CR tightness and preferably colocalized with KMT2A and partners (Fig. 5B). Moreover, counting the colocalization of each gene pair over all single cells, the percentage of CRGs colocalized with both KMT2A and partners is nearly twice that of controls (Fig. 5C, chi-squared test, *P*<0.001).

### CRGs may be involved in the RUNX1-mediated transcription factories

**Fig. 6:**
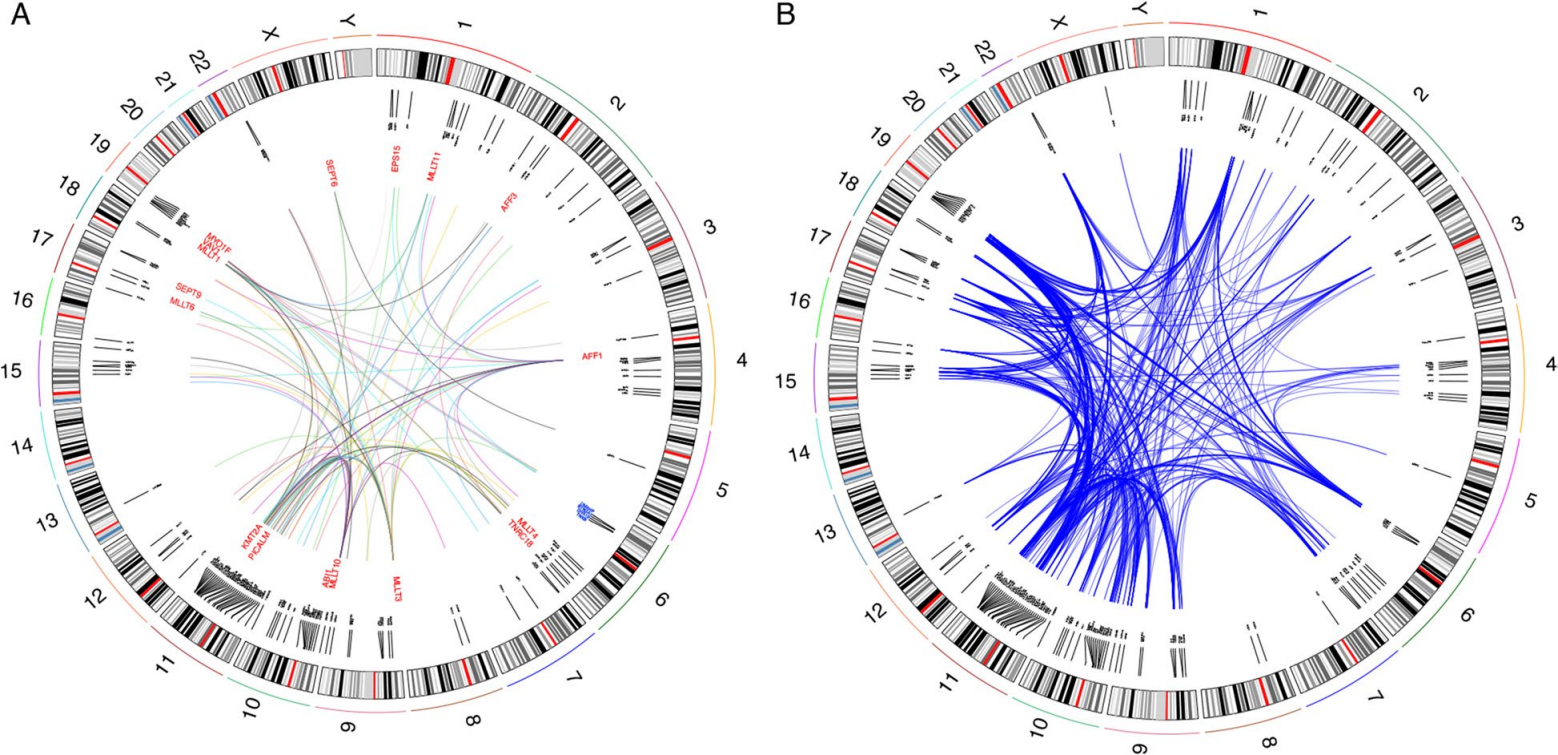
The proximity maps between CRGs and KMT2A fusion partners. **A** Circos plots of CR-generating KMT2A fusions. Genes in the outlier circle (black) indicate CRGs, and genes in the inner circle (red) indicate KMT2A fusion partner genes. **B** The spatial proximity map between different CRGs measured using the single-cell data

We identified 147 CRGs from 153 leukemia samples with only five observed in more than one sample (CEP164, DSCAML1, FXYD2, SIK3, and GRIA4). Four of these reappearing genes are within a 1.68Mb region on chromosome 11. Moreover, DSCAML1, FXYD2, and SIK3 are target genes of transcription factor RUNX1. CRGs form clusters on chromosomes (Fig. 6A). For example, for four fusion partner genes, MLLT3(chr9), MLLT1(chr19), VAL1(chr19), and EPS15(chr1), they all have CRGs located in 6p21 and 6p22 regions (Fig. 6A). Further, different CRGs seem to colocalize in single cells (Fig. 6B). These results suggest that the genomic distribution of CRGs is not random and their involvement in complex rearrangements may be triggered by some common processes.

**Fig. 7:**
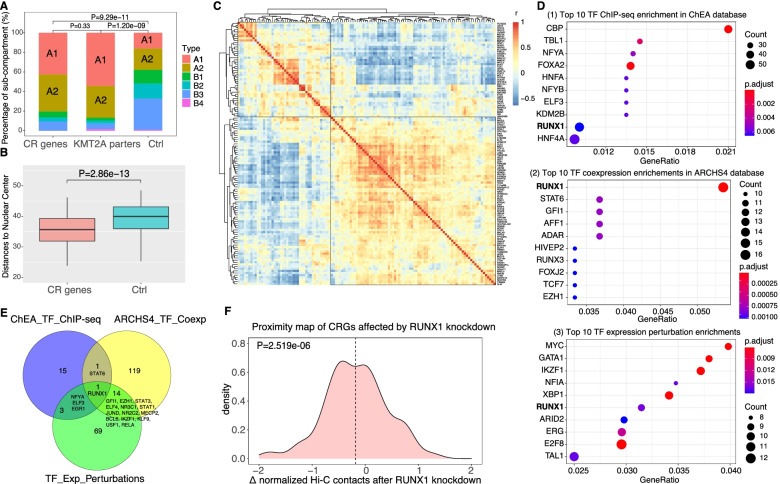
The CRGs may be involved in the RUNX1-mediated transcription factories. **A** The distributions of KMT2A partners, CR genes (CRGs), and control genes in different transcriptional sub-compartments. **B** CRGs are close to the nuclear center than control genes (Wilcoxon rank-sum test). **C** Co-expressions clusters among CRGs. **D** Enrichments of CRGs in the targets of TFs based on three different databases. **E** Intersections of enriched TFs among the three databases. **F** The distribution (density plot) of the distance changes for colocalized CRG pairs before and after RUNX1 knockdown

It was reported that transcription factories (distinct nuclear regions for nascent RNA productions by assembling critical regulatory factors [24, 25]) may contribute to the gene fusions in leukemia [26]. For example, MLLT3 (AF9) and MLLT10 (AF10) shared the same transcription factory with KMT2A [26]. Therefore, we suspect that CRGs get involved into complex rearrangements via transcription factories. In line with this speculation, we found that CRGs are more often located in active A1 and A2 sub-compartments (Fig. 7A, *P*=9.29e−11) and in the interior region of the nucleus (Fig. 7B, *P*=2.86e−13). Moreover, CRGs form co-expressions clusters (Fig. 7C), suggesting the clusters may be transcribed together.

Next, we try to examine whether CRGs are regulated by common transcriptional factors. To do so, we first chose CRG pairs having EuD<=15 in at least three single-cell Hi-C samples and then constructed a network with these pairs. We obtained 126 such genes. We performed enrichment analysis using Enrichr [22] to find enriched transcriptional factors based on three regulation databases [27–29] (see the “Methods” section). Each database provides several enriched transcription factors (Fig. 7D), and RUNX1 (AML1) is the only one provided by all the three (Fig. 7D, E). These results suggest that RUNX1 may be an important factor to form transcription factory and bring CRGs together. In line with this idea, RUNX1 knockdown resulted in significantly fewer contacts among CRGs in MCF7 cell lines (Fig. 7F, *P*=2.519e−06).

### Chromatin loop structures and active transcriptions may drive DNA breakages in CRGs

**Fig. 8:**
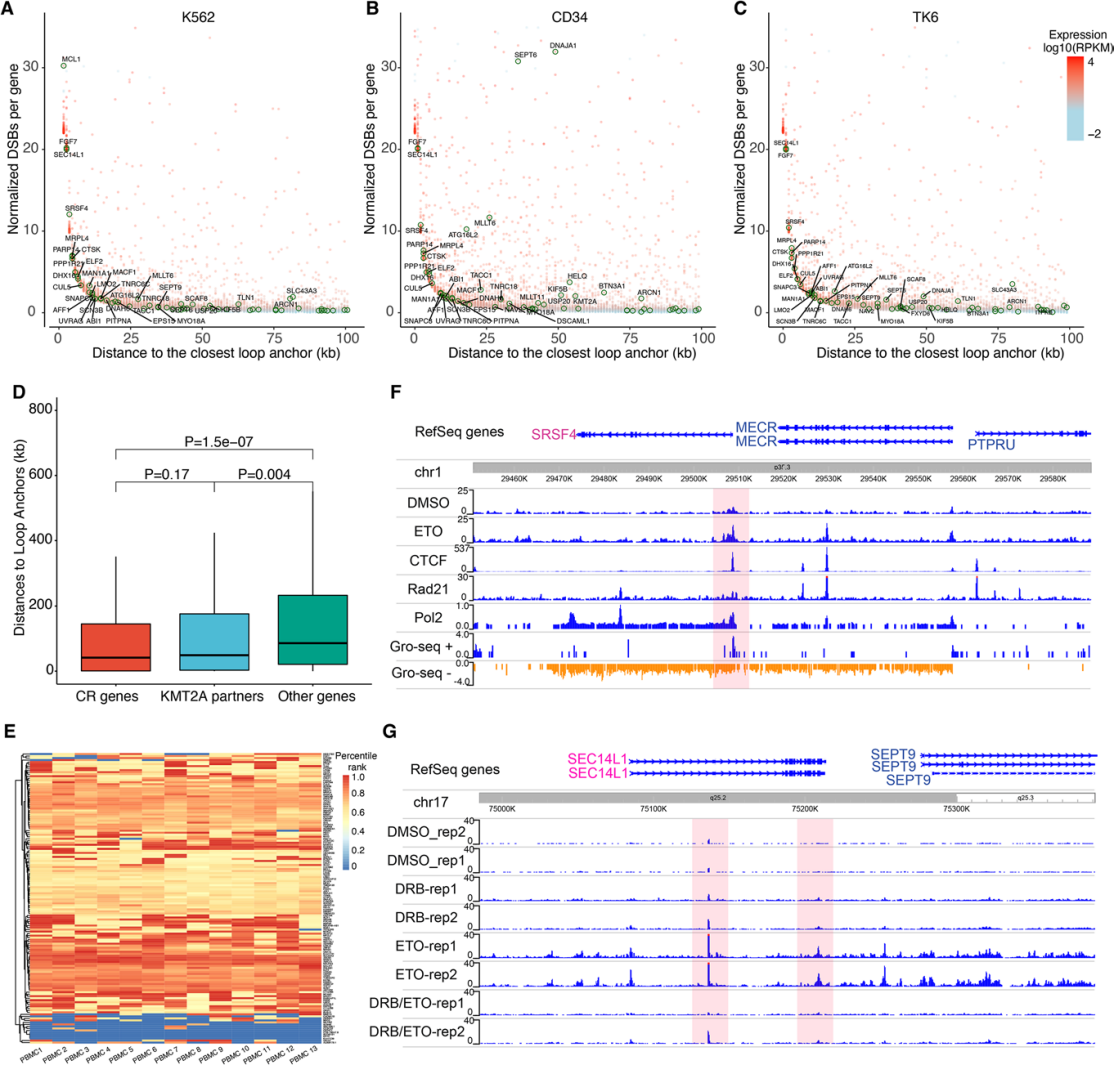
CRGs show active transcriptions, proximity to loop anchors, and high levels DSBs. **A**–**C** The relationship between DBS frequency in a gene and distance to chromosomal loop anchors. In each plot, the expression level is denoted with colors, and CRGs are marked using dark green circles. **D** Comparison of the distances to nearest loop anchors among KMT2A partners, CRGs, and other genes. **E** The heatmap of CRG expression in blood cells, and the gene expression is normalized as percentile in each single cell. **F** CTCF enrichment, transcriptional activity, and ETO-treated breakages in genomic regions around SRSF4 gene in K562 cells. **G** Genome-wide ETO-treated DSB profiles of the SEC14L1 gene in TK6 cells treated with or without DRB (a transcription inhibitor)

If transcription factories bring CRGs and other fusion genes closer, the next question is how DNA breakages occur to form fusions. It was reported that simultaneous breakage and erroneous DNA repair of several genes within the same transcription factory could generate canonical fusions, such as RUNX1-ETO [30]. Moreover, breakages of KMT2A and partners were associated with transcription and chromatin loop structures [20]. Therefore, we hypothesize that breakages and illegitimate ligations of multiple genes in the same transcription factories may underlie CRs and fusions. We tested this hypothesis by examining the relationships between ETO-treated DNA breakages, distances to loop anchors, and transcriptions of CRGs in three hematopoietic cell lines (TK6 cells, K562 cells, and CD34 + cells) using public datasets [20]. Like KMT2A fusion partner genes [20], most CRGs, such as SRSF4, SEC14L1, and FGF7, are subject to high levels of ETO-induced DSBs, high expressions, and adjacent loop anchors in all three cell lines (Fig. 8A–C). CRGs are significantly closer to the loop anchors (Fig. 8D) and among the top highly expressed genes (Fig. 8E). Moreover, ETO-treated K562 cells showed increased sBLISS signals (indicating the levels of DSBs) at the promoter-proximal regions of active CRGs with nascent RNA expressions such as SRSF4 (Fig. 8F), SEC14L1, and PARP14. These genes also were occupied by CTCF and RAD21 and had increased Pol II occupancy at the promoter-proximal regions (Fig. 8F). Disruption of transcriptions by DRB in TK6 cells resulted in decreased DSBs in CRGs with high expressions, such as PARP14 genes (Fig. 8G), SRSF4, and SEC14L1 (Additional file 1: Fig. S5), further supporting that ETO-induced DSBs depend on transcriptions.

## Discussion

In this study, we employed single-cell Hi-C data to investigate the 3D genome structure in blood cells. Compared to the traditional method FISH, Hi-C provides both higher resolution and throughput in evaluating 3D structures. Also, the Hi-C data are highly consistent with the results from FISH. For example, a recent FISH-based study revealed the spatial distances between KMT2A and four partner genes: MLLT1, AFF1, MLLT4, and MLLT3 [20]. Our Hi-C results match the results very well. In addition, single-cell Hi-C can evaluate the spatial locations of many genes simultaneously, providing an opportunity to study all KMT2A partners and CRGs at the same time. To our knowledge, our study is among the first to report how interactions between multiple CRGs and KMT2A fusions are spatially organized in a diploid single cell [14, 15]. Although well consistent with bulk Hi-C (Additional file 1: Fig. S6), single-cell Hi-C can provide a picture of 3D genome structure of each cell, allowing one to see spatial locations of multiple genes. For example, in PBMC cell 18, seven fusion partner genes are colocalized with KMT2A, and in PBMC cell 10, MLLT3 and MLLT10 are colocalized with KMT2A.

Using the single-cell Hi-C data and classifying genes based on their statuses in the COSMIC database, we found that leukemia fusion genes tended to colocalize in normal blood cells, with most fusions incorporating KMT2A. The result was further confirmed by using the SPRITE data. These results support the contact first model, which states that fusion partners tend to colocalize in normal precursor cells before translocation [10]. Interestingly, the colocalizations of leukemia fusion partners are stronger than that of solid tumor fusion partners. This is expected because the Hi-C data are from blood cells, which resemble better the precursor cells of leukemia. This implies that the Hi-C data from precursor cells of solid tumors will reveal a reverse pattern. This explanation is consistent with previous reports that 3D genome structure may contribute to various fusion types in different cancer types [15, 31, 32].

By studying the genes related to complex rearrangements (CRGs), which account for a significant fraction of KMT2A rearrangements [33], we found that the spatial locations of CRGs also support the contact first model. Further, CRGs are enriched in actively transcribed regions, tend to be co-expressed, and are enriched in the targets of transcriptional factors such as RUNX1. These observations made us to suspect that transcription may have brought CRGs together, facilitating their fusions. The suspicion is consistent with the knowledge that transcriptions are highly coordinated and often co-regulated by the same TFs [34], and also consistent with reported associations between KMT2A fusion formations and transcriptions [20]. It was also reported that 2–3% of KMT2A alleles undergoing transcription are spatially close to MLLT3 (AF9) or AFF1 (AF4) and shared transcription factories (specific regions in the nucleus with a microenvironment for active transcription [35]) [26]. Among all significant transcriptional factors, RUNX1 stands out, supported by multiple datasets. The transcriptional factor RUNX1 (AML1) is a master hematopoietic transcription factor and binds to the core element of many enhancers and promoters. The protein encoded by RUNX1 gene represents the alpha subunit of CBF and is thought to be involved in the development of normal hematopoiesis [36]. Therefore, the active transcription of many genes by RUNX1 may increase the chances of fusion of its target genes in blood cells, consistent with that top 30 KMT2A partners were enriched in the targets of RUNX1. Further, transcription factories are dynamic as genes getting in or out of them [37], which may create opportunities to fuse different genes, consistent with the observation of cellular heterogeneity of 3D genome structures among single cells. RUNX1 foci in living cells can exist for more than 30 min and are spatially constrained, but their components are dynamic [24], which may partly explain the big variety of KMT2A fusion partners and CRGs.

The formation of complex rearrangement is a multistep process and starts with the simultaneous occurrence of DSBs in multiple chromosomal regions [8]. Cleavage of hotspots in KMT2A and fusion partners by TOP2 was proposed to trigger the molecular events leading to KMT2A translocations and fusions [33, 38]. Besides spatial colocalizations, most CRGs showed high levels of ETO-induced DSBs, high expressions, and adjacent loop anchors in blood cell lines. These results support the hypothesis that complex rearrangements may be associated with the collapse of transcription factories, including co-regulated genes [1, 39]. The breakages of a transcription factory with dynamic components [24] might partially account for the high heterogeneity of CRGs observed in different leukemia genomes.

## Conclusion

Using the single-cell Hi-C and other data, we demonstrated that the leukemia fusion partner genes tend to be in close proximity in normal blood cells. Our results also suggest that complex rearrangement-associated genes (CRGs) are near transcription factories and their breakages depend on transcription. These results together propose a model that spatial proximity of partner genes and transcription factories may have contributed significantly to leukemia complex rearrangements and oncogenic fusions. Given that the mechanisms underlying complex rearrangements and fusions for different cancer types seem to be tissue specific [3, 30], it is interesting to see how robust this model is for other tumor types.

## Methods

### Single-cell diploid Hi-C datasets

We used public diploid single-cell Hi-C data [18] from the GEO database (accession number GSE117876), which includes 17 single cells from GM12878 (a female human lymphoblastoid cell line) and 18 PBMC cells (several different cell types). There is a median of 1.04 million contacts per single cell. Most cells were in the G1 or G0 phase of the cell cycle.

To ensure the quality of the data used in our analyses, we excluded six GM12878 samples, which contain large chromosomal regions (>10Mb) without any contacts (possibly technical artifacts) (see Table S1 in reference [18]). Finally, the Hi-C data from 11 GM12878 and 18 PBMC single cells were used in our study. The cells include 14 T-lymphocytes, 12 B-lymphocytes, and 3 myeloid cells, providing the opportunity to examine different 3D genome structures among different cell types.

The single-cell Hi-C data were analyzed using the Dip-C algorithm by the original study [18] to construct the diploid genomes at the 20-kb resolution, assuming that two alleles would typically contact different partners and unknown haplotypes can be inferred from neighboring contacts. We adopted the final version of 3D structure models, with the suffix “impute3.round4.clean.3dg,” which contain the 3D localization (x, y, z) of each 20kb bin in the nucleus. The particle model was used to build the single-cell diploid 3D genomes, and each particle represented 20 kb of chromatin with a radius of ~100 nm [18]. The upper axis limit was about 50~60 Euclidean distances (50~60 *100 nm=5~6μm) [18], which agrees with previous reports that the human cell nucleus encloses 46 chromosomes is ~5μm in radius (10 μm in diameter) [40, 41].

### Mapping genes involved in oncogenic fusions to 20kb bins

A list of 297 curated oncogenic fusions was downloaded from the COSMIC database (cancer.sanger.ac.uk) [19]. KMT2A-related complex rearrangements were extracted from a previous study [4]. To get the 3D locations, we mapped each gene to the 20kb bins based on the gene’s genomic coordinates and use the location of the associated bin to represent the 3D location of that gene. When computing the distance between two genes in each cell, we took the minimum distance between any two alleles (paternal or maternal) of the two genes. When applicable, the average or minimum distance over the 29 single-cell samples was computed for each gene pair.

### Control gene pairs

To assess the statistical significance of the distances of fusion gene pairs, we generated control gene pairs as follows: for each fusion gene, we randomly picked genes on different chromosomes to generate control gene pairs, so each control gene pair contains one fusion gene and a random gene from a different chromosome.

### Complex rearrangements (CRs) and complex rearrangement-related genes (CRGs)

The complex rearrangements (CRs) of KMT2A fusions (in 232 patients) were obtained from a previous report (Table S12 in reference [4]). In total, there are 19 different fusion partner genes in these CRs, with the following three genes being most frequent, MLLT10 (62), AFF1 (62), and MLLT3 (32). Besides KMT2A and partners, each complex rearrangement also involves one extra gene, and these extra genes are termed complex rearrangement-related genes (CRGs). Only CRGs located on different chromosomes from KMT2A and corresponding partners are used in our analyses.

### Calculating CR tightness

Similar to a previous study [42], we used the CR tightness score to quantify the spatial tightness among CRG, KMT2A, and partner gene in each complex rearrangement in the cell nucleus. The closer these genes are, the smaller the CR tightness score. Mathematically, the CR tightness of a complex rearrangement is calculated using the following formula:

$${\mathrm{T}}_{\mathrm{G}}=\min \left(\sum \left({\mathrm{EuD}}_{\mathrm{k}-\mathrm{p}},{\mathrm{EuD}}_{\mathrm{k}-\mathrm{c}},{\mathrm{EuD}}_{\mathrm{p}-\mathrm{c}}\right)\ k,p,c\ \mathrm{over}\ \mathrm{maternal}\ \mathrm{allele},\mathrm{paternal}\ \mathrm{allele}\right)$$

where EuD_k-p_, EuD_k-c_, and EuD_p-c_ denote the spatial distances for gene pairs KMT2A-partner, KMT2A-CRG, and partner-CRG, respectively. Since each gene has both maternal and paternal alleles, the considered distances include all combinations of maternal and paternal alleles.

To measure the statistical significance of T_G_, for each complex rearrangement, we generated control set as follows: keep the gene KMT2A and partner gene, and choose a random gene from a different chromosome to replace CRG. Comparisons between CRGs and corresponding control genes are computed by using Wilcoxon rank-sum test in R.

### Transcriptional regulation enrichments

To test enrichment of gene set in the targets of transcriptional factors, we used the Enrichr Transcription module [22], which integrates several common databases, including CHEA and ENCODE ARCHS4 TFs Coexp, and TF Perturbations Followed by Expression [27–29]. Adjusted *P*-value was set at 0.05 as the significant level. The target genes of RUNX1 were obtained from Harmonizome [43]. RNA-seq datasets of peripheral blood mononuclear cells (PBMCs) from 13 individuals were downloaded from the GEO database (accession number GSE107011) and used for gene co-expression analysis [44].

### Hi-C of RUNX1 knockdown MCF cells

The Hi-C normalized data with RUNX1 knockdown in MCF7 cell lines were downloaded from the GEO database (GSE75070) [45].

### Datasets of ChIP-seq, GRO-seq, sBLISS and chromosomal loops

Processed ChIP-seq datasets (in bigwig format) for CTCF, Rad21, and Pol2 of K562 were downloaded from ENCODE [46]. Processed K562 GRO-seq data (bigwig) were downloaded from GEO (GSM1480325). Suspension-cell BLISS (sBLISS) can identify DSBs at nucleotide resolution across the genome. Processed sBLISS data (bigwig and bed formats) for K562, TK6, and CD34+ were downloaded from GEO (GSE121742). The above datasets were visualized with the WashU EpiGenome Browser [47]. Sub-compartment annotations of GM12878 and chromatin loops of K562 were downloaded from GSE63525.

### FISH and quantification of FISH images

GM12878 cell line was purchased from the Shanghai Bluefcell company. FISH probes of KMT2A-ELL and KMT2A-MLLT3 were purchased from the Shanghai Long Island Antibody company. The FISH probes of BCR-ABL1 were purchased from the Guangzhou Anbiping Medical Company. FISH experiments were also performed by Guangzhou Anbiping Medical Company according to the manufacturers’ protocols. The slides were imaged with oil immersion objective on LEICA DMi8 (Leica Camera Company, Germany). The spatial distances between every two signals and the volumes of nuclei were measured with the help of the software Fiji (a distribution of ImageJ) [48].

### Statistical tests

We calculated *p*-values by comparing different groups using Wilcoxon rank-sum test or Student’s *t*-test in R language. The effect size is measured by cohensD function in lsr package. ANOVA is performed by *aov* function in R language. Bartlett’s test (bartlett.test function in R) is used to test if *k* samples have equal variances and Anderson–Darling test R (ad.test function in R) was used for testing for normality.

### Gene aliases of common KMT2A fusion genes

HUGO gene nomenclature have changed over the past years and we use the latest gene nomenclature throughout the text, and these genes with their aliases are listed below: KMT2A (MLL); AFF1 (AF4); AFF3(LAF4); AFF4 (AF5); MLLT1 (ENL); MLLT3 (AF9); MLLT4 (AF6); MLLT6 (AF17); MLLT10 (AF10); and MLLT11 (AF1Q).

## Supplementary Information

Additional file 1; Figure S1. The genomic locations of fusion partners across the genome. Figure S2. Comparison of spatial distances between leukemia fusion gene pairs (from TumorFusions) and control. Figure S3. Comparisons between FISH-based results and single-cell -based results for common KMT2A partners. Figure S4. Comparisons of spatial distances of fusion gene pairs associated with ALL and AML. Figure S5. ETO-induced DSB profiles around other CRGs in TK6 cells. Figure S6. Analysis of bulk Hi-C data from GM12878 and K562 cell lines.

Additional file 2: Table S1. Curated oncogenic fusions from the COSMIC and TumorFusions databases.

Additional file 3. Review history.

## Data Availability

The data used in this study are all publicly available and listed in the following table: **Table:** 

**Data**	**Identifier**	**Source**
Single-cell Hi-C	GSE117876	[49]
RNA-seq of PBMCs	GSE107011	[50]
Hi-C of RUNX1-knockdown MCF7 cells	GSE75070	[51]
ChIP-seq for CTCF, Rad21 and Pol2 in K562	GSE31477	[52]
GRO-seq for K562	GSE60454	[53]
sBLISS for K562, TK6, and CD34+	GSE121742	[54]
Sub-compartment of GM12878	GSE63525	[55]
Chromatin loops of K562	GSE63525	[55] Microscopy images in the study are also accessible at Figshare [56].
