# Supplementary material for: Single-cell diploid Hi-C reveals the role of spatial aggregations in complex rearrangements and KMT2A fusions in leukemia
Source: Genome Biol. 2022 Aug 9;23:173. doi: 10.1186/s13059-022-02740-9 (PMC9361544; doi:10.1186/s13059-022-02740-9)
Supplement: Supplementary file 1 — Additional file 1; Figure S1. The genomic locations of fusion partners across the genome. Figure S2. Comparison of spatial distances between leukemia fusion gene pairs (from TumorFusions) and control. Figure S3. Comparisons between FISH-based results and single-cell -based results for common KMT2A partners. Figure S4. Comparisons of spatial distances of fusion gene pairs associated with ALL and AML. Figure S5. ETO-induced DSB profiles around other CRGs in TK6 cells. Figure S6. Analysis of bulk Hi-C data from GM12878 and K562 cell lines. [file 13059_2022_2740_MOESM1_ESM.pptx]

## Slide 1
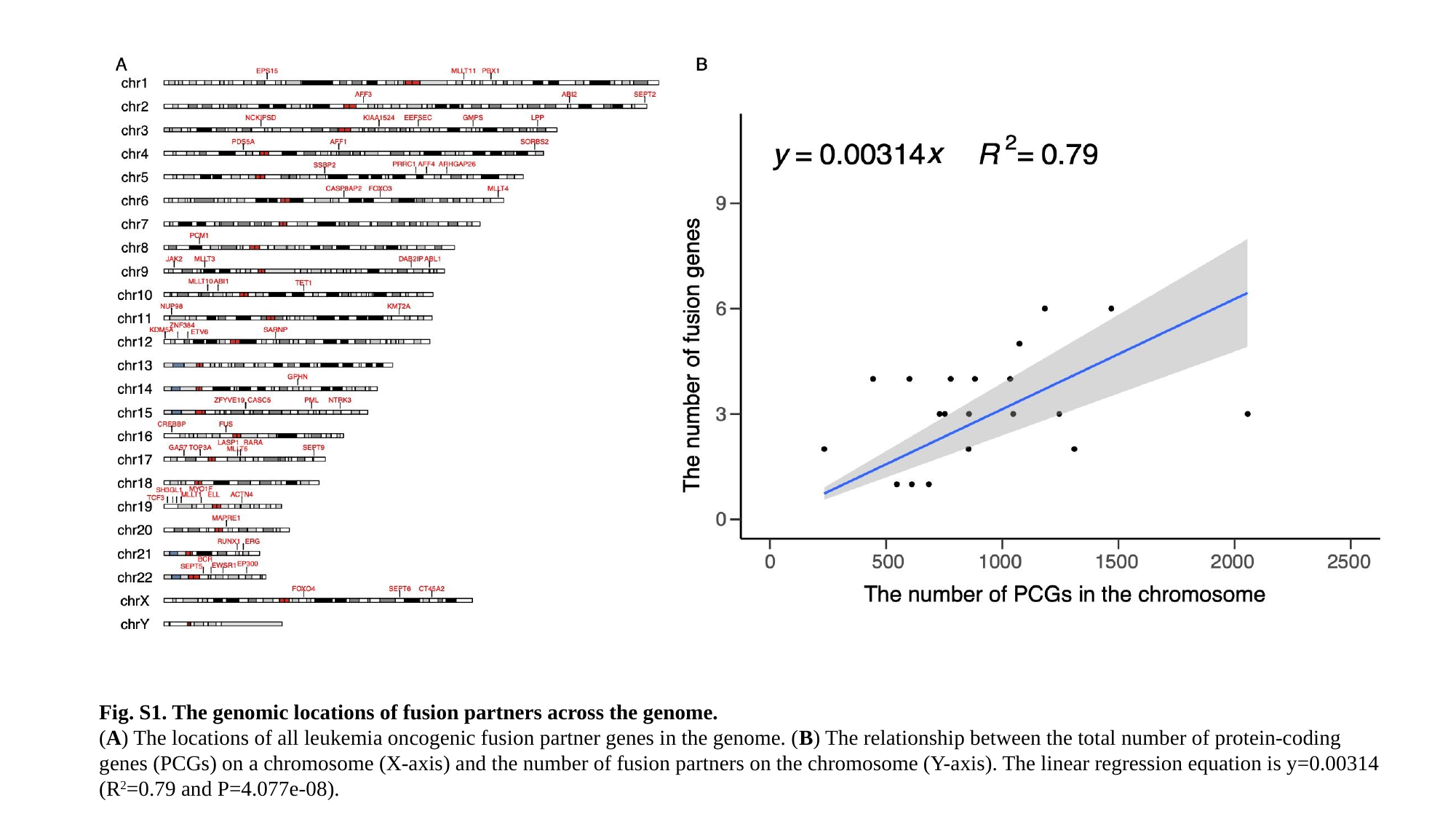

Fig. S1. The genomic locations of fusion partners across the genome.
(A) The locations of all leukemia oncogenic fusion partner genes in the genome. (B) The relationship between the total number of protein-coding genes (PCGs) on a chromosome (X-axis) and the number of fusion partners on the chromosome (Y-axis). The linear regression equation is y=0.00314 (R2=0.79 and P=4.077e-08).

## Slide 2
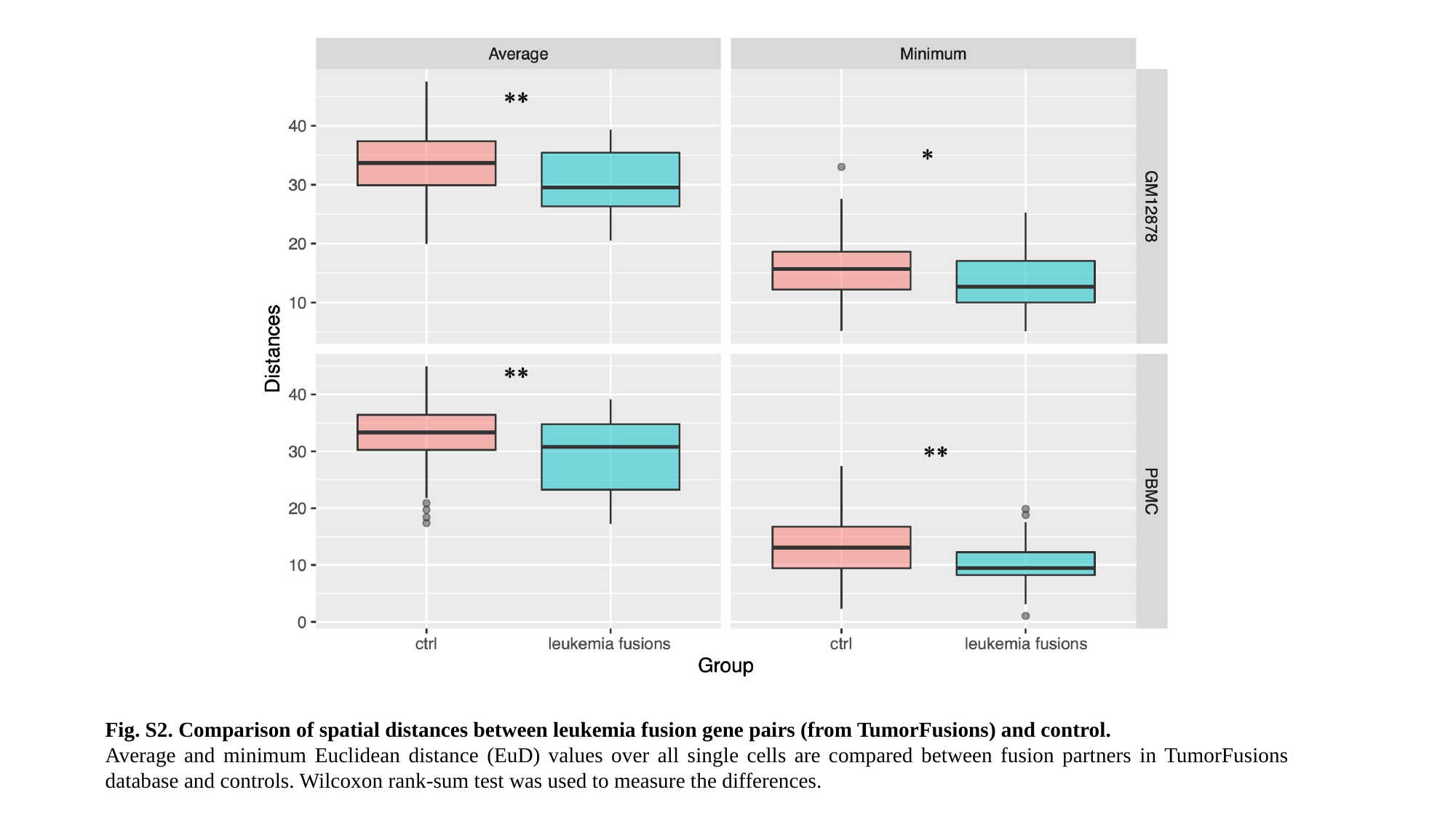

Fig. S2. Comparison of spatial distances between leukemia fusion gene pairs (from TumorFusions) and control.
Average and minimum Euclidean distance (EuD) values over all single cells are compared between fusion partners in TumorFusions database and controls. Wilcoxon rank-sum test was used to measure the differences.

## Slide 3
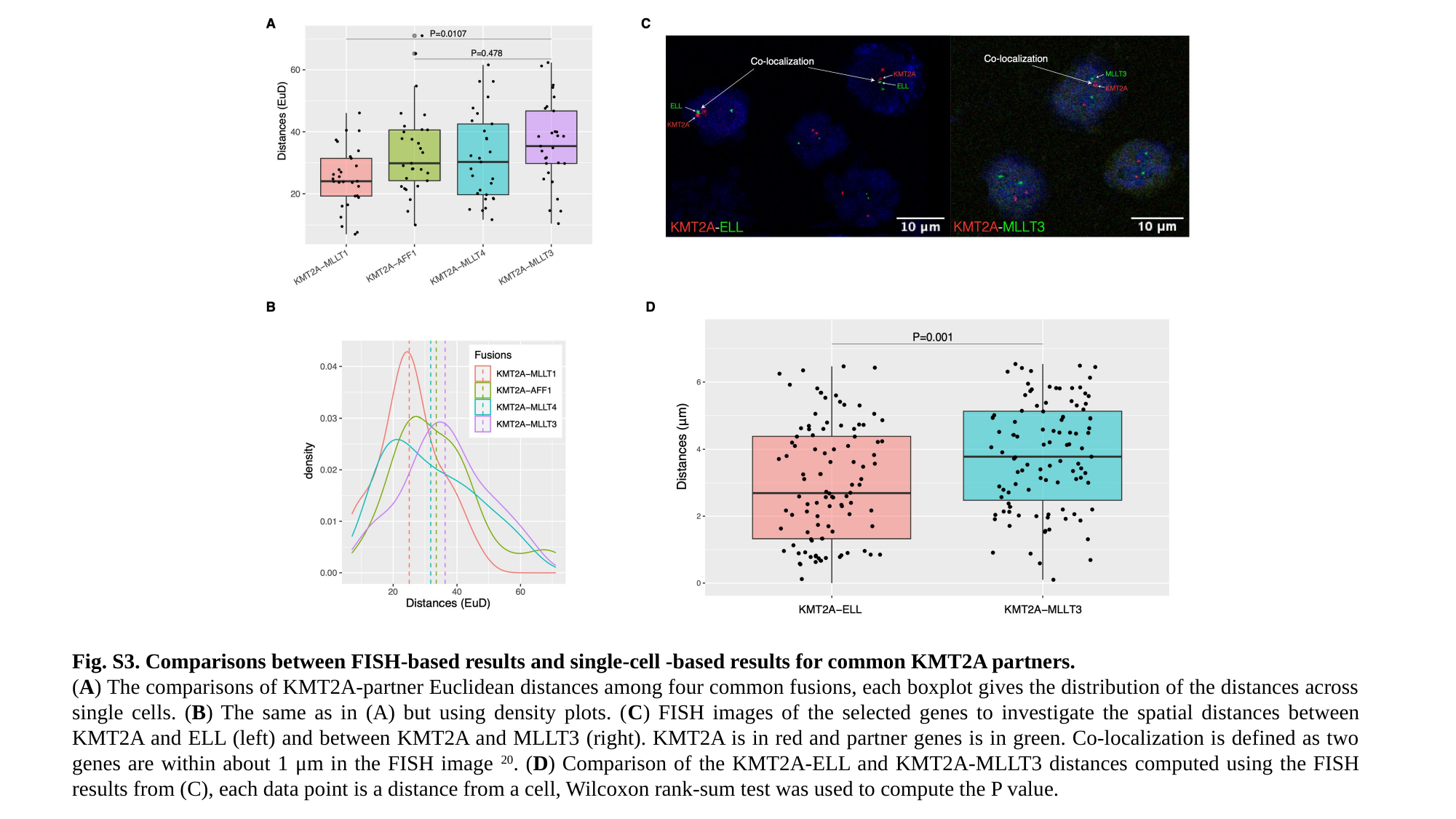

Fig. S3. Comparisons between FISH-based results and single-cell -based results for common KMT2A partners.
(A) The comparisons of KMT2A-partner Euclidean distances among four common fusions, each boxplot gives the distribution of the distances across single cells. (B) The same as in (A) but using density plots. (C) FISH images of the selected genes to investigate the spatial distances between KMT2A and ELL (left) and between KMT2A and MLLT3 (right). KMT2A is in red and partner genes is in green. Co-localization is defined as two genes are within about 1 μm in the FISH image 20. (D) Comparison of the KMT2A-ELL and KMT2A-MLLT3 distances computed using the FISH results from (C), each data point is a distance from a cell, Wilcoxon rank-sum test was used to compute the P value.

## Slide 4
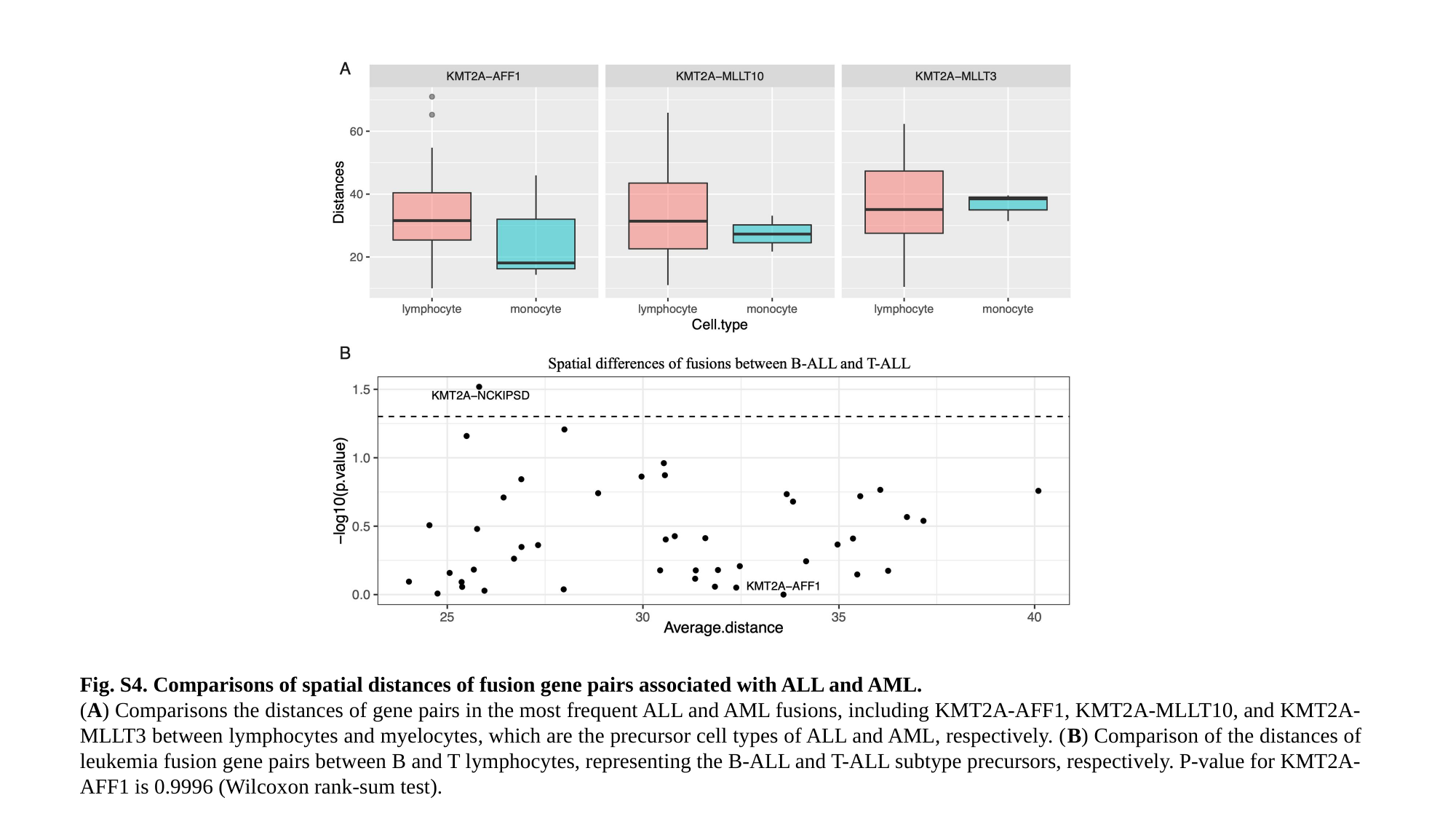

Fig. S4. Comparisons of spatial distances of fusion gene pairs associated with ALL and AML.
(A) Comparisons the distances of gene pairs in the most frequent ALL and AML fusions, including KMT2A-AFF1, KMT2A-MLLT10, and KMT2A-MLLT3 between lymphocytes and myelocytes, which are the precursor cell types of ALL and AML, respectively. (B) Comparison of the distances of leukemia fusion gene pairs between B and T lymphocytes, representing the B-ALL and T-ALL subtype precursors, respectively. P-value for KMT2A-AFF1 is 0.9996 (Wilcoxon rank-sum test).

## Slide 5
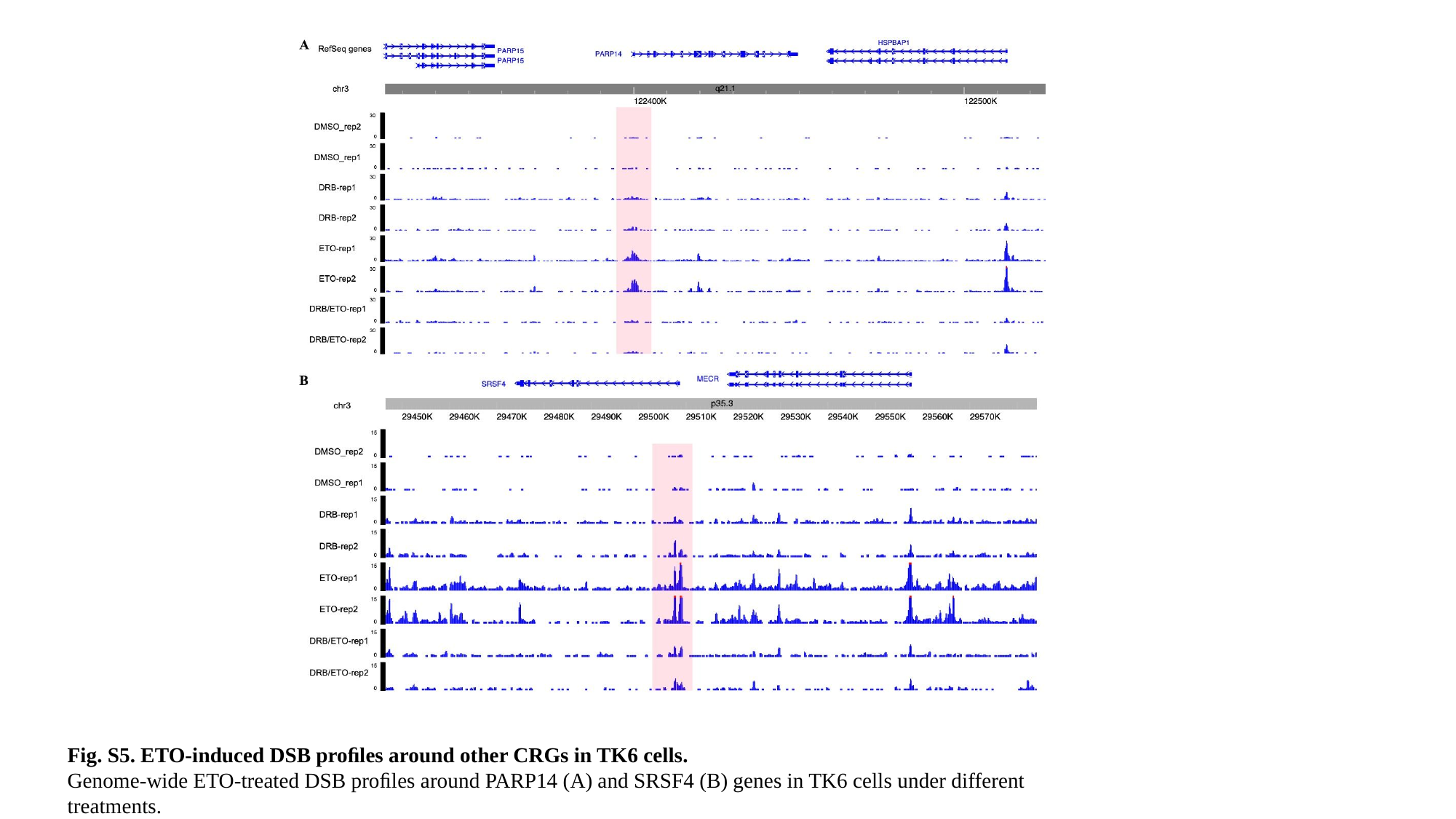

Fig. S5. ETO-induced DSB proﬁles around other CRGs in TK6 cells.
Genome-wide ETO-treated DSB proﬁles around PARP14 (A) and SRSF4 (B) genes in TK6 cells under different treatments.

## Slide 6
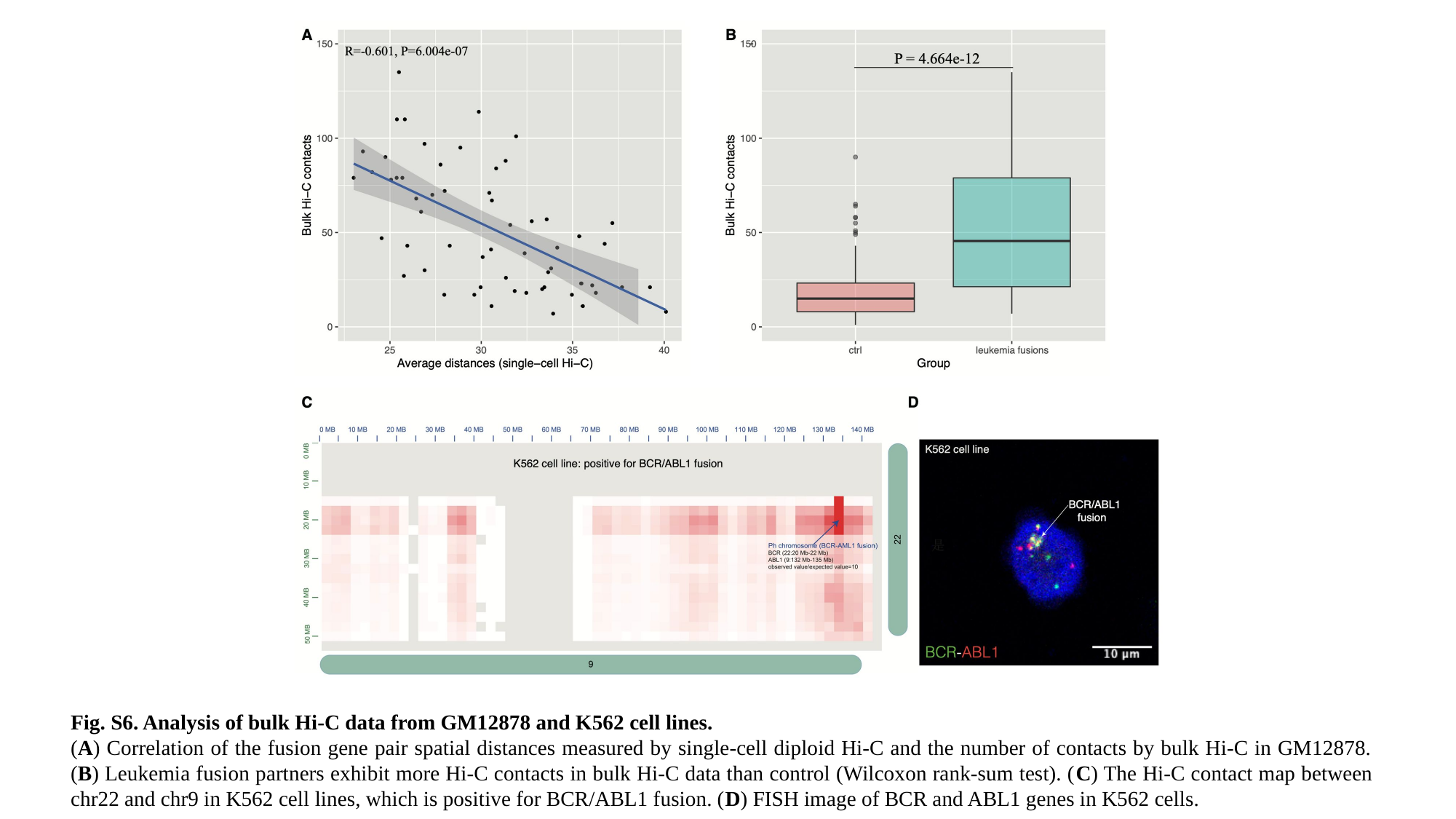

Fig. S6. Analysis of bulk Hi-C data from GM12878 and K562 cell lines.
(A) Correlation of the fusion gene pair spatial distances measured by single-cell diploid Hi-C and the number of contacts by bulk Hi-C in GM12878. (B) Leukemia fusion partners exhibit more Hi-C contacts in bulk Hi-C data than control (Wilcoxon rank-sum test). (C) The Hi-C contact map between chr22 and chr9 in K562 cell lines, which is positive for BCR/ABL1 fusion. (D) FISH image of BCR and ABL1 genes in K562 cells.
