# Supplementary material for: Single-cell diploid Hi-C reveals the role of spatial aggregations in complex rearrangements and KMT2A fusions in leukemia
Source: Genome Biol. 2022 Aug 9;23:173. doi: 10.1186/s13059-022-02740-9 (PMC9361544; doi:10.1186/s13059-022-02740-9)
Supplement: Supplementary file 3 — Additional file 3. Review history. [file 13059_2022_2740_MOESM3_ESM.docx]

Review History

**First round of review**

**Reviewer 1**

**Were you able to assess all statistics in the manuscript, including the appropriateness of statistical tests used?**

No, I do not feel adequately qualified to assess the statistics.

**Comments to author:**

In this manuscript, the authors tried to reanalyze the published single-cell diploid Hi-C data to characterize the relationship between spatial locations of fused genes and complex rearrangement in leukemia. This is somewhat innovative as there is no previous study to demonstrate the relationship between spatial proximity and gene fusion and complex rearrangement from single-cell Hi-C data. Based on their analyses, they claimed that the spatial organizations of KMT2A partners and CRGs may contribute to complex rearrangement generation and KMT2A fusions in leukemia. The results seem a bit trivial as numerous studies have shown similar results. The authors further tried to propose a model to explain the co-expression cluster formation from the perspective of spatial interactions of the CRGs. However, some of the analyses cannot support the authors' conclusions and some statistical comparisons seem to be unreasonable. Several aspects of the manuscript need to be clarified and refined.

Major comments:

1. The contribution of this work should be further clarified. The advantages of single-cell data are not fully reflected in this manuscript.

2. P7, 125-127: Why intra-chromosomal fusions should be removed? What are the possible effects of tissue heterogeneity in intra-chromosomal fusions?

3. P8, 144: What are the solid tumor fusions? The solid tumor fusions should be clearly defined or listed in the supplementary table.

4. P8, 144: As the authors described in the Methods section, the controls were composed of random pairs of bins on different chromosomes. However, the distribution of distances between two random points in 3D space is distinct from that of distances between a fixed point and random points. So I don't think the controls here are reasonable. The authors should compare the distribution of distances between a particular gene and its fusion partners to that of distances between the gene and random genes.

5. P8, 154-156: "Co-localization ratio" is undefined. And the authors reported that "…ranked top 2 fusions (Fig.1C), which suggests that spatial proximity may play an important role in these fusions". Only two fusions are too insufficient to support this speculation. More evidence and details should be showed here.

6. P9-P10, 175-182: What about the statistical significances of the comparisons in Supplementary Figure 2? The differences between the distance distributions of the four groups in Supplementary Figure 2A and 2B seem to be very significant, which is not consistent with the FISH results showed in Figure 2E in [31] of the references. Is there any explanation for this?

7. P11, 207-208: The authors reported that "Transcription factors (TFs)-mediated regulation activities may drive colocalizations and translocations of KMT2A and partners". I think this claim lacks evidence. The authors should be more cautious when drawing conclusions.

8. P12, 218-223: What is the control in Figure 3B? If it is composed of random gene pairs, the result is biased. I don't think the GSEA of fusion partners can support the claim "KMT2A and fusion partners were close to each other in nuclei". I think "spatial proximity" is more appropriate than "3D structure" and "3D genome structure" in this paragraph.

9. P13-P14, 255-257: The authors reported that "These results suggest that spatial proximity under a certain threshold is a requisite, …", what is the threshold means here? Prevalence or something else?

10. In section 5, I don't think the CR tightness of CRGs showed significant difference with control in Figure 5D. Other comparison methods should be considered.

11. P15, 292: Figure 7B cannot support the conclusion that CRGs preferred to be located in the interior region of the nucleus. Comparison with control is needed at least.

12. P16, 302-303: What is the statistical effect size of comparison in Figure 7F? The boxplot seems to make no difference.

13. P16, 305-308: The authors proposed that KMT2A fusion partners and CRGs preferred to be colocalized in the same transcription factory. This should be directly verified according to the definition of transcription factory (1 μm). Moreover, the statement here seems weird as the contextual relevance is weak.

14. P17, 325: What are the "three features"?

15. P25, 484: The CR tightness formula is hard to understand. It should be carefully checked.

Minor comments:

1. There are many places where the citation number is placed after the punctuation mark, for example, line 44, 45, 47, 52, 70, …

2. P3, 52: Grammatical error, "…, remains is remarkably poorly understood"

3. P7, 134: A period is missing here.

4. P9, 174: In "…AFF1, KMT2A, and MLLT3…", KMT2A here should be MLLT4.

5. P12, 235: "…, in addition to 3 fusions that…", "3" should be written as "three"

6. P14, 259: "KMT2A Fusions…" should be "KMT2A fusions"

7. P14, 263: "KMT2A related CRGs were obtained from a previous study", the previous study should be cited.

8. P16, 298: "Three commonly used transcriptional regulation databases…", the databases should be cited.

9. P17, 316: "…high levels breakages…" should be "high level breakages"

10. P20, 389: "transcription transcriptions" seems weird.

11. P21, 404-406: The experiments should be cited.

12. The meaning of the color of the node in Figure 2H should be described.

**Reviewer 2**

**Were you able to assess all statistics in the manuscript, including the appropriateness of statistical tests used?**

Yes, and I have assessed the statistics in my report.

**Comments to author:**

In this paper, Xing et al. studied the 3D genome organization of chromatin variation in leukemia with published omics datasets. The research is based on correlation analysis, which lacks causality analysis, and there are several drawbacks that should be improved before it can be published.

1. In this study, only 297 oncogenic fusions from COSMIC (92 intro-chromosomal and 205 inter-chromosomal) were used for analysis. The number of oncogenic fusions is less than the reported, making it impossible to get results with general statistical significance.

https://tumorfusions.org/

2. There are dynamic changes of 3D genome structure at the location of oncogenic fusions. It can not predict the 3D genome structure of oncogenic fusions with the Hi-C data from normal karyotype cells (i.e., GM12878 and PBMC in this study).

https://www.ncbi.nlm.nih.gov/pmc/articles/PMC7138649/

https://www.nature.com/articles/s41467-021-24044-5

3. When using the inter-chromatin Hi-C data, it's necessary to determine how many accurate signals are in the data.

4. How about the 3D genome structure of intro-chromosomal fusions?

5. What is the relationship between fusions and 3D genome structures such as compartment, TAD, and loops?

**Reviewer 3**

**Were you able to assess all statistics in the manuscript, including the appropriateness of statistical tests used?**

No, I do not feel adequately qualified to assess the statistics.

**Comments to author:**

Chromosome rearrangements producing aberrant fusion genes are common across many cancer types and are particularly prevalent in hematological malignancies. Recurrent fusion genes that occur at high frequency have raised several potential models for their origins. In this manuscript, Xing et al. test the "contact-first" model which posits that genes that recurrently fuse are spatially positioned close to one another in three-dimensional space in the genome. By interrogating previously published single-cell Hi-C data, Xing et al. analyzed the distance of gene pairs from 58 leukemia-specific inter-chromosomal gene fusions obtained from COSMIC compared to controls (gene fusions specific for solid tumors) in GM12878 lymphoblastoid cells and peripheral blood mononuclear cells (PBMCs). The authors provide cell type-specific evidence supporting the contact-first hypothesis of recurrent hematological gene fusions. Although this is a resourceful study that combines multiple datasets and methods to test this hypothesis, there are several concerns that should be addressed prior to publication.

The authors argue that bulk Hi-C utilizing ensemble averaging is insufficient given that tumors arise from a single cell, providing the authors' rationale to explore single cell Hi-C data. However, the sample sizes here for single-cell Hi-C analyses are small and limited to only 11 GM12878 cells and 18 PBMC samples (29 cells in total). This precludes the authors from finding direct and consistent evidence supporting robust spatial contacts of specific gene pairs within multiple cells. Additionally, it is unclear how the 11 GM12878 cells were selected for analysis. Figure 2B lists these Cells 2, 3, 5, etc. Were Cells 1, 4, etc. excluded, and if so, what is the rationale for their exclusion?

The authors should validate some of these findings by interphase FISH using publicly available GM12878 cells, which will allow them to measure spatial distances within hundreds of individual cells. For example, Figure 2B shows high heterogeneity of KMT2A interactions at the single cell level. What are the spatial distances between KMT2A and top colocalization partners in GM12878 nuclei, such as CASC5, ELL, EP300, etc., compared to those that colocalize less frequently/strongly (or not all) in single cell Hi-C data?

The title of the manuscript is much too strong as the data does not directly support the idea that spatial aggregations drive chromosomal rearrangements. Although the data presented here support the idea that recurrent fusion genes are positioned more closely to one another in the genome, these data are largely correlative without direct evidence that they are more susceptible to actual rearrangements. The authors should consider a more appropriate title based on the observations described here.

The authors should consider implementing a direct comparison between bulk Hi-C and single cell Hi-C. Would using a bulk Hi-C approach lead to the same conclusions? Other sequencing methods in the paper are performed in bulk (e.g., ChIP-seq and GRO-seq). Additionally, the authors should consider leveraging single-cell Hi-C data from cells with known fusion genes as positive controls for identifying interactions at the single cell level.

Minor concerns:

The distance calculation used is based on the average or minimum Euclidean distance, but most graphs do not indicate which value is used for distance.

The paper could use some improvement in the presentation of of the figures. Figure legends should include a description of the statistical analysis used to determine significance. Axes of figures can be simplified and made uniform across all figures (e.g., capitalization inconsistencies, axis of Fig 4b is not consistent with other Average Distance axes in the same figure). Misspelling of correlations in Fig 2C. Page 11 line 200 is missing a citation. Page 14 line 260. PDR6C does not match Figure 5a. Page 15 line 289 is missing the word factories after the first word.

**Authors Response**

**Point-by-point responses to the reviewers’ comments:**

Reviewer #1:

In this manuscript, the authors tried to reanalyze the published single-cell diploid Hi-C data to characterize the relationship between spatial locations of fused genes and complex rearrangement in leukemia. This is somewhat innovative as there is no previous study to demonstrate the relationship between spatial proximity and gene fusion and complex rearrangement from single-cell Hi-C data. Based on their analyses, they claimed that the spatial organizations of KMT2A partners and CRGs may contribute to complex rearrangement generation and KMT2A fusions in leukemia. The results seem a bit trivial as numerous studies have shown similar results. The authors further tried to propose a model to explain the co-expression cluster formation from the perspective of spatial interactions of the CRGs. However, some of the analyses cannot support the authors' conclusions and some statistical comparisons seem to be unreasonable. Several aspects of the manuscript need to be clarified and refined.

Major comments:

1. The contribution of this work should be further clarified. The advantages of single-cell data are not fully reflected in this manuscript.

*Reply: Thanks for the comment. Compared to FISH, single-cell Hi-C has higher resolution and throughput, and compared to bulk Hi-C, single-cell Hi-C provides opportunities to see 3D genome structures in single cells and thus to observe multiple fusion genes colocalization in a single cell (Fig. 2A-2C). Following the comment, we have emphasized these advantages in multiple places [e.g. P4,73-80; P15,286-298].*

2. P7, 125-127: Why intra-chromosomal fusions should be removed? What are the possible effects of tissue heterogeneity in intra-chromosomal fusions?

*Reply: In this study, we used only inter-chromosomal fusions for two reasons: (1) 83% of the leukemia fusions reported in the COSMIC database are inter-chromosomal, and (2) some of the intra-chromosomal fusions involve genes in close linear genomic distance, and the Hi-C accuracy of such close gene pairs is low. We have revised our manuscript to add these reasons [P5,91-95].*

3. P8, 144: What are the solid tumor fusions? The solid tumor fusions should be clearly defined or listed in the supplementary table.

*Reply: The solid tumor fusions were also obtained from the COSMIC database and have been listed in supplementary table 1 (sheet: 138 inter solid tumor fusions).*

4. P8, 144: As the authors described in the Methods section, the controls were composed of random pairs of bins on different chromosomes. However, the distribution of distances between two random points in 3D space is distinct from that of distances between a fixed point and random points. So I don't think the controls here are reasonable. The authors should compare the distribution of distances between a particular gene and its fusion partners to that of distances between the gene and random genes.

*Reply: This is an excellent suggestion, and we agree that the reviewer suggested approach is more appropriate than our old one. Following this suggestion, we have generated controls by randomly choosing gene pairs from each fusion gene and other genes (see the updated Methods section Control gene pairs (P20, 387-391)). With this update, our conclusion remains unchanged (e.g., Fig. 1B). For your reference, we also list the statistical P values derived from comparing the distances of fusion gene pairs and control (for both old and updated versions). As you can see in the below table, the results are not changed much.*

| **Statistics** | **P-value (old)** | **P-value (updated)** |
| --- | --- | --- |
| Average EuD (All cells) | 1.463047e-14 | 1.473687e-07 |
| Minimum EuD (All cells) | 8.845057e-11 | 1.02162e-09 |
| Average EuD (GM12878) | 2.083986e-10 | 6.984276e-05 |
| Minimum EuD (GM12878) | 2.904318e-08 | 9.510566e-06 |
| Average EuD (GM12878) | 1.095828e-12 | 1.503844e-07 |
| Minimum EuD (GM12878) | 7.144348e-09 | 2.816928e-09 |

5. P8, 154-156: "Co-localization ratio" is undefined. And the authors reported that "…ranked top 2 fusions (Fig.1C), which suggests that spatial proximity may play an important role in these fusions". Only two fusions are too insufficient to support this speculation. More evidence and details should be showed here.

*Reply: Co-localization ratio is defined as the ratio of cells in which a gene pair is colocalized, and we have added the information in P7, 120-121.*

*As for “…ranked top 2 fusions”, we meant that Philadelphia chromosome (Ph) fusion BCR-ABL1 (co-localization Ratio=34.9%) and KMT2A-ELL (co-localization Ratio=27.6%) are the top 2 fusions based on colocalization ratio. Accordingly, we have rephrased our text at P7, 123-127. We apologize for the confusion.*

6. P9-P10, 175-182: What about the statistical significances of the comparisons in Supplementary Figure 2? The differences between the distance distributions of the four groups in Supplementary Figure 2A and 2B seem to be very significant, which is not consistent with the FISH results showed in Figure 2E in [31] of the references. Is there any explanation for this?

*Reply: This is a great point. Actually, we performed ANOVA analysis and found that the gene-pair distances among the four fusions were significantly different (P=0.0107, Supplementary Fig.3A). However, no significant difference was detected when comparing the following three fusions: KMT2A-AFF1, KMT2A-MLLT4 and KMT2A-MLLT3 (P=0.478, Supplementary Fig.3A). Further, pairwise comparisons supports that MLLT1 is closer to KMT2A in proximity than MLLT4 (P=0.0096), AFF1 (P=0.04) and AF9 (P=0.0007); the result is consistent with the FISH results in Figure 2E of reference ^1^. We have added the statistical test results in the revised manuscript in P8, 139-141. The boxplots may be a bit visually confusing and we overlay data points on the boxplots to show the variation in each group.*

7. P11, 207-208: The authors reported that "Transcription factors (TFs)-mediated regulation activities may drive colocalizations and translocations of KMT2A and partners". I think this claim lacks evidence. The authors should be more cautious when drawing conclusions.

*Reply: We agree that this was a bold conclusion. However, we do have several pieces of evidence to support the conclusion: (1) the CRGs and fusion partner genes are enriched in the targets of several transcriptional factors, (2) these genes are co-expressed, (3) CRGs are enriched in active transcriptional regions and chromosomal loop anchors, and (4) the occurrence of double-strand breaks in these genes are transcription-dependent. We agree with the reviewer that the evidence may not be very strong given that our analyses are computational, and thus we have weakened our statements to caution readers (Results section 5 and 6).*

8. P12, 218-223: What is the control in Figure 3B? If it is composed of random gene pairs, the result is biased. I don't think the GSEA of fusion partners can support the claim "KMT2A and fusion partners were close to each other in nuclei". I think "spatial proximity" is more appropriate than "3D structure" and "3D genome structure" in this paragraph.

*Reply: As answered above in Fig.1B, we have adopted the reviewer suggested method to generate control gene pairs. With the updated control, we found that the conclusion still holds (Fig.3B, P=1.807e-5). Thanks for the suggestion again.*

*As for "KMT2A and fusion partners were close to each other in nuclei", we apologize for the confusion. Actually, we meant "KMT2A fusion partner genes are significantly enriched in genes that are spatially close to KMT2A gene in the nuclei", and have updated the text (P9, 173-176).*

*We agree that “spatial proximity” is more appropriate here and revised the text accordingly.*

9. P13-P14, 255-257: The authors reported that "These results suggest that spatial proximity under a certain threshold is a requisite, …", what is the threshold means here? Prevalence or something else?

*Reply: We used a wrong and confusing term “under a certain threshold” , which should be “spatial proximity”. We have corrected the statement.*

10. In section 5, I don't think the CR tightness of CRGs showed significant difference with control in Figure 5D. Other comparison methods should be considered.

*Reply: Agree. Figure 5D was a not a good piece of evidence and is removed in the revised manuscript. Instead, we use Fig.5B to show that the CRGs are significantly closer to KMT2A and AFF1 than control (P=0.023). Thanks for pointing this out.*

11. P15, 292: Figure 7B cannot support the conclusion that CRGs preferred to be located in the interior region of the nucleus. Comparison with control is needed at least.

*Reply: It might be that our statement was not clear. As shown in the updated Fig. 7B, CRGs are significantly closer to the nuclear center (P=2.86e-13).*

12. P16, 302-303: What is the statistical effect size of comparison in Figure 7F? The boxplot seems to make no difference.

*Reply: The difference was statistically significant but the effect size was small Cohen’s d = 0.038. However, this was caused by using all gene pairs among the CRGs, but only some pairs are actually colocalized. Therefore, in this revision, we restrict our analysis into 269 CRG pairs, which are colocalized based on the single-cell Hi-C data. With that, Figure 7F shows that RUNX1 knockdown resulted in significantly fewer contacts among CRGs in MCF7 cell lines (P=2.519e-06, Wilcoxon rank sum test), and the effect size is Cohen’s d = 0.305. We also replaced boxplot of two groups with density plot of group differences to make the differences easier to visualize.*

13. P16, 305-308: The authors proposed that KMT2A fusion partners and CRGs preferred to be colocalized in the same transcription factory. This should be directly verified according to the definition of transcription factory (1 μm). Moreover, the statement here seems weird as the contextual relevance is weak.

*Reply: I think that our statements were not accurate. Yes, the scale of transcription factory is 1 μm. We have removed the inaccurate statements.*

14. P17, 325: What are the "three features"?

*Reply: The three features are high levels of ETO-treated DSBs, high expression, and adjacent loop anchors. To clarify this, we have rephrased the sentence to “most CRGs such as SRSF4, SEC14L1, and FGF7 shared high levels of ETO-induced DSBs, high expressions, and adjacent loop anchors in all three cell lines (Fig.8A-C).” as in P14, 272-275.*

15. P25, 484: The CR tightness formula is hard to understand. It should be carefully checked.

*Reply: Our original description may not be clear. We have updated the text to make the definition of CR tightness clear. Please see P21, 400-410.*

Minor comments:

1. There are many places where the citation number is placed after the punctuation mark, for example, line 44, 45, 47, 52, 70, …

*Reply: We have corrected these errors. Thanks.*

2. P3, 52: Grammatical error, "…, remains is remarkably poorly understood"

*Reply: We have fixed this error by removing ‘is’. (P3, 53-54).*

3. P7, 134: A period is missing here.

*Reply: Added. (P6, 102).*

4. P9, 174: In "…AFF1, KMT2A, and MLLT3…", KMT2A here should be MLLT4.

*Reply: Thanks for point out this error. We have corrected it now.*

5. P12, 235: "…, in addition to 3 fusions that…", "3" should be written as "three"

*Reply: changed.*

6. P14, 259: "KMT2A Fusions…" should be "KMT2A fusions"

*Reply: fixed.*

7. P14, 263: "KMT2A related CRGs were obtained from a previous study", the previous study should be cited.

*Reply: The paper is cited now. (P12, 221).*

8. P16, 298: "Three commonly used transcriptional regulation databases…", the databases should be cited.

*Reply: the citations have been added in P16, 302.*

9. P17, 316: "…high levels breakages…" should be "high level breakages"

*Reply: the typo is fixed now (P13, 254).*

10. P20, 389: "transcription transcriptions" seems weird.

*Reply: it should be “transcription factories”, and the text has been corrected.*

11. P21, 404-406: The experiments should be cited.

*Reply: The studies of these experiments have been cited now (P17, 321).*

12. The meaning of the color of the node in Figure 2H should be described.

*Reply: A darker color for a node means more connections in the network as in P29, 580-581.*

*Many thanks to the reviewer for carefully reading our manuscript and for providing so many valuable comments and suggestions.*

Reviewer #2:

In this paper, Xing et al. studied the 3D genome organization of chromatin variation in leukemia with published omics datasets. The research is based on correlation analysis, which lacks causality analysis, and there are several drawbacks that should be improved before it can be published.

1. In this study, only 297 oncogenic fusions from COSMIC (92 intro-chromosomal and 205 inter-chromosomal) were used for analysis. The number of oncogenic fusions is less than the reported, making it impossible to get results with general statistical significance. <https://tumorfusions.org/>

*Reply: Before we started the project, we tried to choose the best data source for oncogenic fusions. Given that the COSMIC database provides the most comprehensive and accurate annotations for somatic mutations (including fusions), we thus chose the COSMIC database.*

*Using the TumorFusions database, we obtained 62 fusions (34 inter-chromosomal and 28 intra-chromosomal), listed in Supplementary Table 1 (sheet: fusions in TumorFusion database). Using these fusions, we reached the same conclusion that fusion partner genes are spatially closer than control (Supplementary Figure 2). This result reinforces our conclusion. Thanks for referring us to the database.*

2. There are dynamic changes of 3D genome structure at the location of oncogenic fusions. It can not predict the 3D genome structure of oncogenic fusions with the Hi-C data from normal karyotype cells (i.e., GM12878 and PBMC in this study). <https://www.ncbi.nlm.nih.gov/pmc/articles/PMC7138649/> <https://www.nature.com/articles/s41467-021-24044-5>.

*Reply: We completely agree with the reviewer that 3D genome structure in a cell change dynamically. As shown in our single cell data, different cells of the same cell type also show colocalization of different set of genes. In this study, we aim to understand what may have contributed to the formation of oncogenic fusions in tumor precursor cells, other than to investigate the 3D genome structures in tumor cells. As the ‘contact first’ hypothesis states, a fusion may occur when two genes are in close spatial proximity. Since tumor cells are evolved from normal cells and oncogenic fusions are important steps, and that is why we focused on normal blood cells and tried to understand the leukemia fusions.*

3. When using the inter-chromatin Hi-C data, it's necessary to determine how many accurate signals are in the data.

*Reply: We used the following three approaches to ensure our data have high quality: (1) the used single-cell 3D genome structure was constructed with the algorithm Dip-C by the original study ^2^; the study showed ~96% imputation accuracy for inter-chromosomal contacts; (2) we removed six samples which have large chromosomal regions (>10Mb) without contacts (possibly technical artifacts); (3) Hi-C is not very effective to detect too far-apart contacts, so we supplemented our analyses by using the data generated with SPRITE – a technique to detect long-distance contacts; with the SPRITE data, we observed the same pattern. These together ensure our results are reliable.*

4. How about the 3D genome structure of intro-chromosomal fusions?

*Reply: As mentioned in another response, this study focuses on inter-chromosomal fusions for two reasons: (1) 83% of the leukemia fusions reported in the COSMIC database are inter-chromosomal, and (2) some of the intra-chromosomal fusions involve genes in close linear genomic distance, and the Hi-C accuracy of such close gene pairs is low. We have revised our manuscript to add these reasons [P5, 91-95].*

5. What is the relationship between fusions and 3D genome structures such as compartment, TAD, and loops?

*Reply: We analyzed the relationships between fusions and compartments and chromosomal loops, and found that (1) KMT2A fusion partner genes and CRGs tend to be located in active sub-compartments (Fig.7A, P=9.29e-11 and P=1.20e−09), and (2) KMT2A fusion partner genes and CRGs were significantly closer to chromosomal loop anchors (Fig.8D, P=1.5e-07 and P=0.004). We have not analyzed the relationship with TAD yet.*

Reviewer #3:

Chromosome rearrangements producing aberrant fusion genes are common across many cancer types and are particularly prevalent in hematological malignancies. Recurrent fusion genes that occur at high frequency have raised several potential models for their origins. In this manuscript, Xing et al. test the "contact-first" model which posits that genes that recurrently fuse are spatially positioned close to one another in three-dimensional space in the genome. By interrogating previously published single-cell Hi-C data, Xing et al. analyzed the distance of gene pairs from 58 leukemia-specific inter-chromosomal gene fusions obtained from COSMIC compared to controls (gene fusions specific for solid tumors) in GM12878 lymphoblastoid cells and peripheral blood mononuclear cells (PBMCs). The authors provide cell type-specific evidence supporting the contact-first hypothesis of recurrent hematological gene fusions. Although this is a resourceful study that combines multiple datasets and methods to test this hypothesis, there are several concerns that should be addressed prior to publication.

1. The authors argue that bulk Hi-C utilizing ensemble averaging is insufficient given that tumors arise from a single cell, providing the authors' rationale to explore single cell Hi-C data. However, the sample sizes here for single-cell Hi-C analyses are small and limited to only 11 GM12878 cells and 18 PBMC samples (29 cells in total). This precludes the authors from finding direct and consistent evidence supporting robust spatial contacts of specific gene pairs within multiple cells. Additionally, it is unclear how the 11 GM12878 cells were selected for analysis. Figure 2B lists these Cells 2, 3, 5, etc. Were Cells 1, 4, etc. excluded, and if so, what is the rationale for their exclusion?

*Reply: Thanks for the thoughtful comments. To directly test the ‘contact-first’ hypothesis, one should monitor the 3D genome structure changes in a cell over time until fusions occur, then he could tell how often a fusion is due to frequent contacts of those genes in 3D space. In reality, this is hard to achieve.*

*Instead, in this study we reasoned that if ‘contact-first’ hypothesis is correct, we expect to see that the genes in a fusion are spatially close before fusion occurs in tumor precursor cells. For leukemia fusions, we used the 3D genome structures in normal blood cells to infer the 3D genome structures in tumor precursor cells. We know this is not perfect, but this is the best we can do at present.*

*As for Fig 2B, we excluded six GM12878 cells because they contain large chromosomal regions (>10Mb) without any contacts (possibly technical artifacts). We have updated the text to clarify the procedure (P19, 362-365).*

2. The authors should validate some of these findings by interphase FISH using publicly available GM12878 cells, which will allow them to measure spatial distances within hundreds of individual cells. For example, Figure 2B shows high heterogeneity of KMT2A interactions at the single cell level. What are the spatial distances between KMT2A and top colocalization partners in GM12878 nuclei, such as CASC5, ELL, EP300, etc., compared to those that colocalize less frequently/strongly (or not all) in single cell Hi-C data?

*Reply: Actually, based on the study publishing the single Hi-C data, Hi-C technique is pretty accurate compared to FISH, so we are confident on the results. However, following the reviewer’s suggestion, we did FISH experiments in GM12878 cells and compared the colocalization between KMT2A and two fusion partner genes: ELL and MLLT3. Based on single-cell Hi-C data, ELL is colocalized with KMT2A more often and closer than MLLT3. And our FISH results are consistent with the Hi-C data, as shown in Supplementary Fig.3C-D.*

*Alternatively, we also used public FISH data to corroborate the Hi-C results. A previous study ^1^ of the frequent KMT2A fusion partner genes based on the FISH technique found that for four fusion partner genes MLLT1, AFF1, MLLT4, and MLLT3, the spatial distance of MLLT1 to KMT2A is much closer than the other three genes. Using the Hi-C data, we confirmed the finding (Supplementary Fig.3A-B, ANOVA test: P=0.0107; Fig.2A). These results together suggest that the Hi-C data accurate enough for us to do statistical analyses.*

3. The title of the manuscript is much too strong as the data does not directly support the idea that spatial aggregations drive chromosomal rearrangements. Although the data presented here support the idea that recurrent fusion genes are positioned more closely to one another in the genome, these data are largely correlative without direct evidence that they are more susceptible to actual rearrangements. The authors should consider a more appropriate title based on the observations described here.

*Reply: We agree, and have changed the title to “Single-cell Diploid Hi-C Reveals that Spatial Aggregations may Contribute to Complex Rearrangements and KMT2A Fusions in Leukemia” (P1, 1-3).*

4. The authors should consider implementing a direct comparison between bulk Hi-C and single cell Hi-C. Would using a bulk Hi-C approach lead to the same conclusions? Other sequencing methods in the paper are performed in bulk (e.g., ChIP-seq and GRO-seq). Additionally, the authors should consider leveraging single-cell Hi-C data from cells with known fusion genes as positive controls for identifying interactions at the single cell level.

*Reply: Thanks for the suggestions. Following the suggestions, we have compared the bulk Hi-C and single-cell Hi-C data, and we also tested a known fusion using FISH to show that the single-cell Hi-C results are reliable.*

*We obtained the bulk Hi-C data for GM12878 from the NCBI GEO database (GSE63525). The bulk Hi-C data measures the number of contacts for gene pairs, so the closer two genes are, the* **more contacts. As** *shown in Supplementary Fig.6A, we do see a negative correlation between the number of bulk Hi-C contacts and the distance of fusion gene pairs measured with single-cell Hi-C (R =-0.601, P=6.004e-07). Further, using the bulk Hi-C data, we could also observe that fusion gene partners are spatially closer than controls (Supplementary Fig.6B, P=4.664e-12). These results reinforce our conclusions derived from the single-cell Hi-C data.*

*As single-cell Hi-C data from cells with known fusion genes is not available, we chose the bulk Hi-C data of K562 cells (positive for BCR-ABL1 fusion) as a positive control and validated the BCR-ABL1 fusion using FISH experiment (Supplementary Fig.6D). K562 cells were human immortalized myelogenous leukemia cell line. We observed a strong contact signal between BCR and ABL1 in the Hi-C contact heatmap (Supplementary Fig.6C). These results together again suggest that the results from the single-cell Hi-C data are reliable.*

Minor concerns:

1. The distance calculation used is based on the average or minimum Euclidean distance, but most graphs do not indicate which value is used for distance.

*Reply: We have updated both figures and legends to clarify which distances are used. Thank you.*

2. The paper could use some improvement in the presentation of of the figures. Figure legends should include a description of the statistical analysis used to determine significance. Axes of figures can be simplified and made uniform across all figures (e.g., capitalization inconsistencies, axis of Fig 4b is not consistent with other Average Distance axes in the same figure). Misspelling of correlations in Fig 2C. Page 11 line 200 is missing a citation. Page 14 line 260. PDR6C does not match Figure 5a. Page 15 line 289 is missing the word factories after the first word.

*Reply: Thanks for these detailed suggestions. We have updated all these errors/typos (see Fig. 2C, 4B, 5A) and improved the manuscript thoroughly, which we believe is more readable now.*

***Reference***

*1. Gothe, H. J. et al. Spatial Chromosome Folding and Active Transcription Drive DNA Fragility and Formation of Oncogenic MLL Translocations. Mol. Cell* ***75****, 267-283.e12 (2019).*

*2. Tan, L., Xing, D., Chang, C.-H., Li, H. & Xie, X. S. Three-dimensional genome structures of single diploid human cells. Science* ***361****, 924–928 (2018).*

**Second round of review**

**Reviewer 1**

In this revised manuscript, the authors carefully addresed some of my questions, but unfortunately, I think some problems still remain unsolved. That is, the conclusions in several subsections of "Results" cannot be supported by their analyses. The authors should be more rigorous in summarizing the results. Moreover, I think the motivation of this work is a little trivial as it's intuitive that gene fusion is conditional on spatial proximity. The contribution of this work to the corresponding community is uncertain as it neither produces data sources nor provides novel discoveries. However, this work showed the importance of spatial proximity in chromosome complex rearrangement (CR), which may inspire the research of disease associated CRs. So, I think it can be considered by Genome Biology if the aforementioned problems are improved. More details of my concerns are listed below:

1. In the first paragraph of the first subsection of "Results", the authors claimed that spatial distances may be a factor to promote fusions only based on the fact that three chromosomes (17, 19, 22) are located in the nuclear center. They showed that the three chromosomes contain more fusion genes, but they ignored that these three chromosomes also have higher gene density.

2. The comparisons of the fusion colocalization ratios between solid tumor and leukemia did not consider the volumes of different cell nuclei.

3. The writing should be further improved.

**Reviewer 2**

Despite some improvements, from my point of view, this study still should improve several issues as follows.

There are multi-scaled chromatin structural differences between lymphocytic leukemia and myeloid leukemia. it’s suggested to compare the spatial aggregation between the fusion genes of lymphocytic leukemia and the fusion genes of myeloid leukemia with the single cells data (14 T-lymphocytes, 12 B-lymphocytes, and 3 myeloid cells).

In the figure1D, the distance between the maternal BCR and ABL1 is larger than the paternal BCR and ABL1. It’s recommended to study and show the distance of all alleles.

In the supplemental-figure3C, it’s recommended to show several co-localization results.

**Reviewer 3**

*The authors have adequately addressed my concerns. I recommend this study for publication in Genome Biology.*

**Authors Response**

**Point-by-point responses to the reviewers’ comments:**

Reviewer #1:

In this revised manuscript, the authors carefully addresed some of my questions, but unfortunately, I think some problems still remain unsolved. That is, the conclusions in several subsections of "Results" cannot be supported by their analyses. The authors should be more rigorous in summarizing the results. Moreover, I think the motivation of this work is a little trivial as it's intuitive that gene fusion is conditional on spatial proximity. The contribution of this work to the corresponding community is uncertain as it neither produces data sources nor provides novel discoveries. However, this work showed the importance of spatial proximity in chromosome complex rearrangement (CR), which may inspire the research of disease associated CRs. So, I think it can be considered by Genome Biology if the aforementioned problems are improved. More details of my concerns are listed below:

1. In the first paragraph of the first subsection of "Results", the authors claimed that spatial distances may be a factor to promote fusions only based on the fact that three chromosomes (17, 19, 22) are located in the nuclear center. They showed that the three chromosomes contain more fusion genes, but they ignored that these three chromosomes also have higher gene density.

*Reply: It is possible that when a chromosome has a higher gene density, it may have larger chances of intra-chromosomal fusions because of smaller average distances between consecutive genes. However, our study is focused on inter-chromosomal fusions only, so chromosomal gene densities should not affect our results.*

*The reviewer’s comment however inspires us to examine the number of genes (specifically protein-coding genes) on each chromosome, because a chromosome with more genes may happen to have more fusion partner genes if inter-chromosomal fusions happen randomly. Therefore, we examined the relationship between the number of fusion partner genes on a chromosome and the number of protein-coding genes on the chromosome. Indeed, we see a positive correlation between these two (Fig. R1, below, shaded area denotes the confidence intervals of predicted means) and that some chromosomes may deviate from expectation based on the number of genes on the chromosomes (those outside the shaded area).*

*In previous version of the manuscript, we stated that the three chromosomes (17, 19, 22) had more fusion partner genes than others, possibly due to their proximity to nuclear center. However, with this new analysis, we see that some other chromosomes also have more fusion genes than expected based on the number of genes on each chromosome, so we think that our original statement isn’t accurate and is thus deleted in the revised manuscript.*

*We also added Fig. R1 as Supplementary Fig. 1B in the manuscript to show the important relationship found here.*

*Thanks for the reviewer’s insightful comment, helping us to interpret the result more accurately.*

***Fig. R1***

2. The comparisons of the fusion colocalization ratios between solid tumor and leukemia did not consider the volumes of different cell nuclei.

*Reply: Actually, in these comparisons the same data set of spatial distances were used (all from the blood cells as described in Methods), so cell nuclei volumes are not a factor.*

*In detail, we extracted fusion gene pairs from databases, and then grouped the gene pairs according to tumor types (i.e., leukemia and solid tumors). Then we computed the colocalization ratio for each gene pair using the blood cell Hi-C data and compared between groups. As seen here, we only used leukemia and solid tumors to annotate the sources of gene fusions and no Hi-C data from leukemia or solid tumor tissues were used.*

*We regret that our original text might not be clear enough regarding this analysis, so we have revised the text to clarify the idea (P6, L111).*

3. The writing should be further improved.

*Reply: We have re-examined the whole manuscript, clarified text and fixed typos. We hope that the new version is more readable.*

**Reviewer 2**

Despite some improvements, from my point of view, this study still should improve several issues as follows.

There are multi-scaled chromatin structural differences between lymphocytic leukemia and myeloid leukemia. it’s suggested to compare the spatial aggregation between the fusion genes of lymphocytic leukemia and the fusion genes of myeloid leukemia with the single cells data (14 T-lymphocytes, 12 B-lymphocytes, and 3 myeloid cells).

*Reply: Thanks for the suggestion. As shown in the original Fig. 4B, we compared the Euclidean distances of KMT2A-partner gene pairs between lymphocytes and myeloid cells, and found no difference, except for 3 gene pairs: KMT2A-ABI1, KMT2A-MLLT4, and KMT2A-SORBS2. However, this analysis did not distinguish the sources of gene fusions: lymphocytic leukemia versus myeloid leukemia, so based on the reviewer’s suggestion, we have improved our analysis as described below.*

*We grouped the fusions based on leukemia types: ALL fusions, AML fusions, and fusions common to both types. And then for each group, we compared the Euclidean distances of the fusion gene pairs between in lymphocytes and in myelocytes. Based on “contact first” hypothesis, we might expect that the ALL fusion gene pairs would show smaller distances in lymphocytes than in myelocytes because lymphocytes may be a better representative of ALL precursors, and vice versa for the AML fusion gene pairs. However, we found no evidence for the expectation, consistent with our original conclusion. We have updated Fig. 4B to show the new analysis and changed the text accordingly (P10, L192).*

In the figure1D, the distance between the maternal BCR and ABL1 is larger than the paternal BCR and ABL1. It’s recommended to study and show the distance of all alleles.

*Reply: In the study, we defined the distance between each gene pair as the minimum distance of any two alleles, regardless of their parental origins, because a fusion of two genes can happen among any combinations of two alleles (paternal-paternal, maternal-maternal, paternal-maternal). In Fig. 1D, it happened that the paternal alleles of BCR and ABL1 are the closest. To clarify the point, we have marked both maternal and paternal alleles of BCR and ABL1 and revised the text accordingly (P6, L105).*

In the supplemental-figure3C, it’s recommended to show several co-localization results.

*Reply: In the revised Supplementary Fig. 3C, we have added some examples of co-localized gene pairs. Thank you for the recommendation.*

**Reviewer 3**

The authors have adequately addressed my concerns. I recommend this study for publication in Genome Biology.

*Reply: Thanks for your constructive comments which have been very helpful in improving our manuscript. We appreciate it.*

**Third round of review**

**Reviewer 1**

*All my previous comments have been addressed.*
